# Coral Reefs at the Northernmost Tip of Borneo: An Assessment of Scleractinian Species Richness Patterns and Benthic Reef Assemblages

**DOI:** 10.1371/journal.pone.0146006

**Published:** 2015-12-31

**Authors:** Zarinah Waheed, Harald G. J. van Mil, Muhammad Ali Syed Hussein, Robecca Jumin, Bobita Golam Ahad, Bert W. Hoeksema

**Affiliations:** 1 Department of Marine Zoology, Naturalis Biodiversity Center, Leiden, The Netherlands; 2 Borneo Marine Research Institute, Universiti Malaysia Sabah, Kota Kinabalu, Sabah, Malaysia; 3 Institute of Biology Leiden, Leiden University, Leiden, The Netherlands; 4 WWF-Malaysia, Kota Kinabalu, Sabah, Malaysia; Ecole normale superieure de Lyon, FRANCE

## Abstract

The coral reefs at the northernmost tip of Sabah, Borneo will be established under a marine protected area: the Tun Mustapha Park (TMP) by the end of 2015. This area is a passage where the Sulu Sea meets the South China Sea and it is situated at the border of the area of maximum marine biodiversity, the Coral Triangle. The TMP includes fringing and patch reefs established on a relatively shallow sea floor. Surveys were carried out to examine features of the coral reefs in terms of scleractinian species richness, and benthic reef assemblages following the Reef Check substrate categories, with emphasis on hard coral cover. Variation in scleractinian diversity was based on the species composition of coral families Fungiidae (n = 39), Agariciidae (n = 30) and Euphylliidae (n = 15). The number of coral species was highest at reefs with a larger depth gradient i.e. at the periphery of the study area and in the deep South Banggi Channel. Average live hard coral cover across the sites was 49%. Only 7% of the examined reefs had > 75% hard coral cover, while the majority of the reef sites were rated fair (51%) and good (38%). Sites with low coral cover and high rubble fragments are evidence of blast fishing, although the observed damage appeared old. Depth was a dominant factor in influencing the coral species composition and benthic reef communities in the TMP. Besides filling in the information gaps regarding species richness and benthic cover for reef areas that were previously without any data, the results of this study together with information that is already available on the coral reefs of TMP will be used to make informed decisions on zoning plans for conservation priorities in the proposed park.

## Introduction

The second largest coral reef area in Sabah, Malaysia, is found at the northernmost tip of Borneo [1] around the Kudat and Bengkoka peninsulas. Its position at the western boundary of the Coral Triangle, which has undergone some shifts over recent years [2–5], implies high marine biodiversity in the area. In addition to extensive coral reefs, large mangrove forests and seagrass beds, this area situated in between the South China Sea and the Sulu Sea functions as an important migratory route for pelagic fish, marine mammals and sea turtles, which also utilize these marine ecosystems as habitat, nesting and feeding grounds [6]. Over 187,000 people live in the main towns of the peninsulas, with almost half of them depending on the marine resources for their livelihood [7–8]. Recognizing the importance of conserving and managing the coral reefs and other marine ecosystems, the Sabah State Government has approved the establishment of this area as a marine protected area (MPA) designated as the Tun Mustapha Park (referred to as TMP hereafter) [9].

Similar to other coral reefs in the region, the reefs of TMP face an array of threats, primarily from unsustainable and destructive fishing methods [10–11]. According to a profile of the ecological, socio-economic aspects and historical trends of reef fisheries at southern Banggi, the largest island in the proposed park [12–13], the reef fisheries are showing signs of early stage Malthusian overfishing, but the productivity of fisheries has not declined to a critical level [14].

Descriptions of the coral reefs in the TMP mainly cover small parts of the islands and reefs [15–17]. Hard coral cover ranged from 4 to 85% between 1996 and 1999 [10], and in southeast Banggi more than 50% of the reefs had “poor” coral cover (< 25% cover) from 1999 to 2002 [18–20] following the criteria developed by the ASEAN-Australia Living Coastal Resources project [21]. Along the east coast of Banggi and the reefs eastwards of Malawali Island, 86% of the reefs had “fair” or “good” coral cover (25%–50% and 50%–75% coral cover, respectively) [22–25]. At the west coast of the Kudat peninsula, mean hard coral cover was around 40% and a majority of the reef sites here were rated “fair” [26–28]. In all these survey reports, blast fishing and overfishing were consistently mentioned as the main threats to the reefs within the TMP.

Very few surveys have been made to document the coral species richness in the TMP. From nine dives and along 28 transects, a total of 273 species of scleractinian corals have been reported around Banggi and the reefs southeast of Malawali [25, 29]. This number seems to be quite low considering TMP’s position between the South China Sea and the Coral Triangle, both of which are large regions with over 550 reef coral species [30].

MPAs or marine reserves have been successful in enhancing the biomass and diversity of overexploited communities [31–34]. MPAs have also shown to be effective in preventing coral loss [35] and aiding in coral recovery [36], though some studies have found otherwise [37–38]. Nevertheless, MPAs can only be successful if the design and function are in line with their goals [39]. In the case of the proposed TMP, the management plans were established with three goals: 1) to eradicate poverty, 2) to ensure suitable development, and 3) to conserve its habitat and threatened species [7–8]. Using a set of MPA design principles developed by stakeholders [40–41] and ecological and socio-economic data compiled from previous surveys and local knowledge, a series of multiple-use zoning plans was created. Data such as key species and habitat features were among other information that were applied to the conservation planning software Marxan with Zones [42], which then identified priority areas for three zones: 1) preservation zones, 2) community-managed zones, and multiple-use zones [8]. The preservation zone is purely for habitat conservation where any form of extractive activities is prohibited, with the exception of permit-approved research. For this zone, the coral reef ecosystems were divided into four ecological regions based on their exposure to wind and currents. In each region, at least 30% of each ecosystem (coral reefs, seagrass and mangroves) and unique features (such as turtle and dugong habitats) required inclusion within the zone [7]. The coral reefs were further categorized into eight reef habitat types defined by reef morphology and exposure [8]. As such, it is important to have information on the reef features and distribution within the TMP in order to optimise the selection process of the potential coral reef sites for the preservation zones.

While some information is available from previous surveys, there are areas within the proposed park that are without any data. In order to address this issue, as well as the need to identify potential reef habitats for the preservation zones, the Tun Mustapha Park Expedition (TMPE) 2012 was organized. The present study was carried out during the expedition and aimed to examine specific features of the coral reefs by focusing on hard coral species richness and benthic assemblages within the TMP. The main objective was to determine the species richness patterns of the scleractinian coral families Fungiidae, Agariciidae and Euphylliidae, which were used as proxy (~ 84 species) for all scleractinian reef corals (> 500 species) [5, 43]. These coral families were selected because they are common on reefs in the Coral Triangle, where they can be found from onshore to offshore reefs and over a wide depth range from shallow reef flats to the lower reef slopes [44–50]. The second objective was to examine the benthic assemblages as a proportion of the reef that is covered by benthos (hard coral, soft coral, sponge, nutrient indicator algae, recently killed coral, rock, rubble, sand, silt, and other substrate), particularly the hard coral component across the study sites. The species richness patterns and benthic communities were assessed in relation to environmental factors: 1) depth (between shallow and deep reef sites), 2) exposure (between sites that are sheltered and exposed to dominant wind-driven waves and currents), and 3) proximity from the mainland. These factors have been found to be important in structuring reef species composition and diversity across coral reef systems (e.g. [45–49, 51–58]) but with varying degrees of influence from one reef system to another. As the resolution of data collection for the coral families and the benthic assemblages were very different, both objectives were treated separately throughout this study.

## Materials and Methods

### Ethics Statement

Research permits were obtained from the Economic Planning Unit, Prime Minister’s Department Malaysia and the Sabah Biodiversity Centre. The surveys were non-destructive and carried out with caution to prevent any damage to the coral reef ecosystem.

### Physical Setting

The proposed park is a corridor where the South China Sea meets the Sulu Sea through the Balabac Strait, which separates north Borneo from the Philippines. The eastern limit of the South China Sea begins at the tip of the Kudat peninsula, Tanjung Simpang Mengayau [variations in spelling = Tanjong Sampanmangio, Tanjung Simpangmangayau], towards the north along the west coast of Balambangan Island, Malaysia and the west coast of Balabac Island, The Philippines. The area to the east of this boundary is defined as the Sulu Sea [59].

The TMP will cover an area of approximately 8,987 km^2^, comprising the coastal areas of the Kudat and Bengkoka peninsulas, and Marudu Bay, which is bounded by the two peninsulas [9]. Coral reefs in the TMP extend over 450 km^2^ [60] and consist of fringing and patch reefs, with banks and shoals situated on a relatively shallow sea floor. Several extensive reef flats can also be found such as the Bankawan Reefs in the northeast of Banggi Island, covering over 100 km^2^. Most of the reefs are established along a gradually sloping substrate before levelling into sandy sea floor. Reefs fringing the coastline are in turbid waters adjacent to a shallow seafloor (≤ 35 m) with gentle slopes that do not have much vertical incline [17]. Moderately steep reef slopes (~ 25–30 degree angle) are found near the Selat Banggi Selatan (referred to as the South Banggi Channel hereafter) towards the south and southeast of the island. A few reef sites in the easternmost and northernmost part of the TMP are also in deeper waters, with much improved water visibility. Owing to the complex morphology of the coastline and islands, and generally shallow depth, there is no clear cross-shelf zonation pattern in the coral reef area.

The northeast monsoon lasts from November to March, and the southwest monsoon prevails from May to September. During the northeast monsoon, when rainfall is heaviest [61], water from the Pacific Ocean enters the Bohol Sea through the Surigao Strait and splits into two branches: one towards Mindoro and the other towards east Sabah. From the latter, the water passes through the Balabac Strait into the South China Sea. In contrast, currents from the South China Sea enter into the Sulu Sea from August to October [62].

### Field sampling

**Fig 1:**
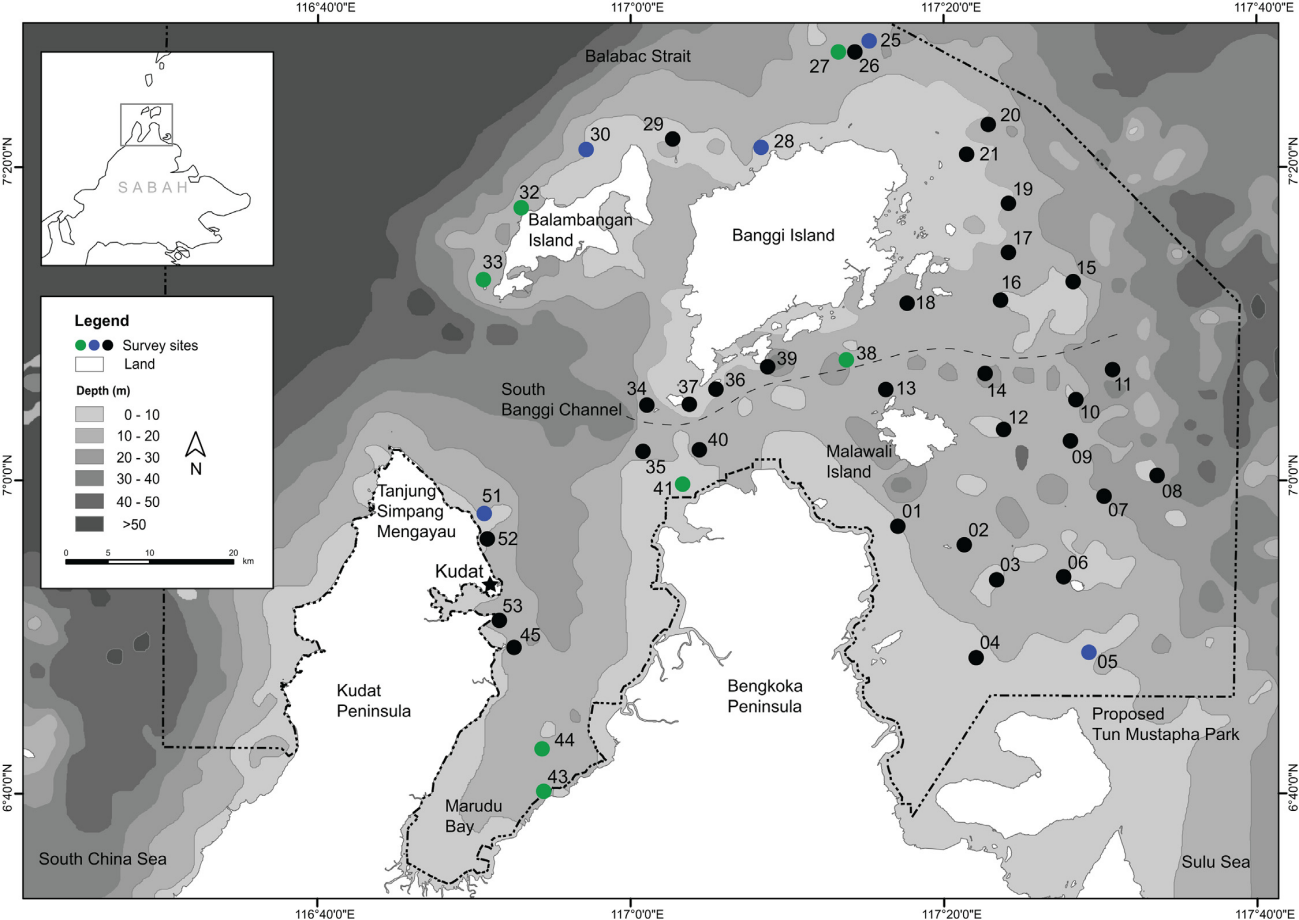
Research area at the northern tip of Borneo. Symbols for data collected at survey sites; green: coral species richness only; blue: benthic community only: black: both. The sites indicated by the black symbol were plotted according to GPS coordinates of the coral species richness surveys (S1 Table). Labels (locality names excluding the proposed Tun Mustapha Park boundary) are given according to British Admiralty Nautical Charts no. 948 and 1654. Selat Banggi Selatan (South Banggi Channel) is indicated by the dashed grey line.

Coral reefs were surveyed during TMPE 2012 in Kudat, Sabah (6°40'– 7°30' N, 116°40’– 117°40’ E) from 6 to 26 September 2012 at coastal and offshore reefs (1–35 m depth). Dives were made at 38 sites for coral species richness surveys and 36 sites for benthic community surveys, situated less than 60 km from the mainland (Fig 1, S1 Table). Sites were selected based on navigation charts (Malaysia Nautical Chart MAL 871, British Admiralty Nautical Charts no. 948 and 1654), reconnaissance dives prior to the expedition, and local knowledge. The selection was intended to be representative of the TMP reef area and include as much habitat diversity as possible, but some areas were excluded from the surveys because of the presence of saltwater crocodiles (*Crocodylus porosus*), particularly at the east and southeast coast of Balambangan Island [63–64]. Several areas were not accessible during the survey due to unfavourable weather conditions with strong winds from the west. Due to this, there was a sampling bias given that the reefs in the west along the coast of the Kudat peninsula were not as fully explored as the reef complex in the east. On several occasions, reefs could not be located due to bad surface visibility accompanied by strong surface currents and waves. Furthermore, only few surveys were possible south of the Kudat town in Marudu Bay owing to poor water visibility caused by wind-generated waves.

### Coral species richness

Coral species incidence data was recorded at each site by adapting the roving diver technique [65–66]. This survey method involved diving along the reef for approximately 60 minutes from the reef base (≤ 35 m depth) to the reef crest or reef flat (~ 1 m depth) while recording target coral species on a slate board. This method gives a better overview of the coral fauna in terms of species presence/absence than the use of belt transects, which is depth-specific and aimed at obtaining species abundance data [46–47, 67]. Each site was surveyed only once, hence no replicates were made. Photographs were taken for each coral species encountered at every site, and specimens that could not be identified in situ were collected for closer inspection and kept in the reference collection of the Borneo Marine Research Institute, Universiti Malaysia Sabah in Kota Kinabalu, Sabah.

Scleractinian coral families Fungiidae, Agariciidae and Euphylliidae were used as proxy for all reef coral species. The fungiids were surveyed at all sites during the present study (n = 38) and nine sites previously surveyed in 2005 (n = 2), 2007 (n = 2) and 2008 (n = 5) (S1 Fig). The identification of this family was primarily based on taxonomic revisions by Hoeksema [68] and Gittenberger et al. [69]. For consistency in the comparisons with previous works, two new members of the Fungiidae, *Cycloseris explanulata* (Van der Horst, 1922) and *C*. *wellsi* (Veron and Pichon, 1980), previously classified as Siderastreidae [70–71] were not included in the survey though both species were present in the area. Data on the Agariciidae and Euphylliidae (sensu Veron [44]) were collected at only 35 sites during the present study and identified based on the taxonomic criteria of Veron [44], Veron and Pichon [72], Dinesen [73], and Ditlev [74].

### Benthic composition

Standard Reef Check methodology [75] was applied to assess the condition of the coral reefs in the TMP. However, data of only the benthic cover are presented here. The benthic communities were quantified at 36 sites and at two depth zones where possible (3–5 m and 8–10 m). In total, 55 transects were surveyed with 33 shallow and 22 deep (mid-reef) sites. For the substrate survey, Reef Check essentially uses the Point Intercept Transect (PIT) method [76], which involves recording the substrate type that lies directly below the transect line at 0.5 m intervals along four 20 m segments of a 100 m transect. A 5 m gap was left in between each segment to create replicates (of four segments) for each transect. The substrate is expressed as mean percentage cover for 10 substrate categories: hard coral, soft coral, sponge, nutrient indicator algae (all algae except coralline, calcareous and turf algae), recently killed coral, rock, rubble, sand, silt and others (substrate not indicated in the previous nine categories) [75].

### Data analysis

In general, the study area was situated in relatively shallow depths, except for sites in the South Banggi Channel and the Mangsee Great Reef. The sites are exposed to winds from the northeast or the southwest, except for sites within Marudu Bay. Each site was in close proximity to an island (land mass), for example 70% of the sites were ≤ 5 km from an island, and only one site (site 15) was > 10 km away. As the distance to the nearest land mass was not variable, we examined the effect of distance from the mainland (Sabah coastline) to the sites. The a priori details for the depth, exposure and distance from the mainland of each site are given in S2 Table. The reef sites surveyed for coral species richness and benthic data from the transects ranged from 0.16 to 5 km apart. The benthic surveys were limited by depth (10 m maximum depth), while a greater depth range was necessary to record as many coral species as possible for the species richness surveys. The reefs are established on a gently sloping substratum, so the deeper slopes were at times a distance away from the shallow parts of the reef, hence creating some distance between both surveys. Thus, direct comparisons of both data types could not be made and the analyses for the coral species richness and benthic communities were made separately.

The coral species incidence data from 47 sites for the fungiids (38 sites from the present study and nines sites from previous surveys) and 35 sites for the agariciid and the euphylliid corals were used for species richness analyses. Species richness estimators were calculated using the software EstimateS [77] to indicate whether the sampling effort had been sufficient in representing the expected total coral species richness in the area [78]. Multivariate analyses were used to examine the coral species richness patterns based on the coral species composition of 35 sites, where data was collected for all three coral families. Similarity profile analysis (SIMPROF) [79] was used to identify the significant clusters of these sites based on the Bray-Curtis similarity measure [80]. A group-averaged hierarchical clustering dendrogram was generated from 1,000 expected and simulated profiles using the R package *clustsig* [81]. The clusters are shown on the map to determine species richness patterns. To further interpret the coral species richness patterns in relation to the environmental conditions (as listed in S2 Table), data of the species richness were regressed across depth, exposure and distance from the mainland using a Generalised Linear Model (GLM) of the Poisson type in R [82] and additional package *car* [83] for model diagnostics.

For the benthic communities, the percentage cover of the substrate categories was averaged for all shallow and deep transects in order to get an overview of the benthic composition in the TMP. The benthic compositions were also visualised by transect and assessed by factors depth and distance from the mainland. Data was then transformed using the box cox transformation in order to meet the assumption of normality. Silt was excluded from subsequent analyses as the percentage cover was extremely low (0.4%, range: 0–4%). Two separate sets of regression analyses were performed to investigate the influence of depth and distance from mainland on the benthic communities. Reef exposure was not examined because the very few transects in the sheltered reef condition prevented any meaningful comparisons between the benthic communities. All statistical analyses were carried out in R and model diagnostics with the *car* package [82–83].

## Results

### Coral species composition and richness patterns

**Fig 2:**
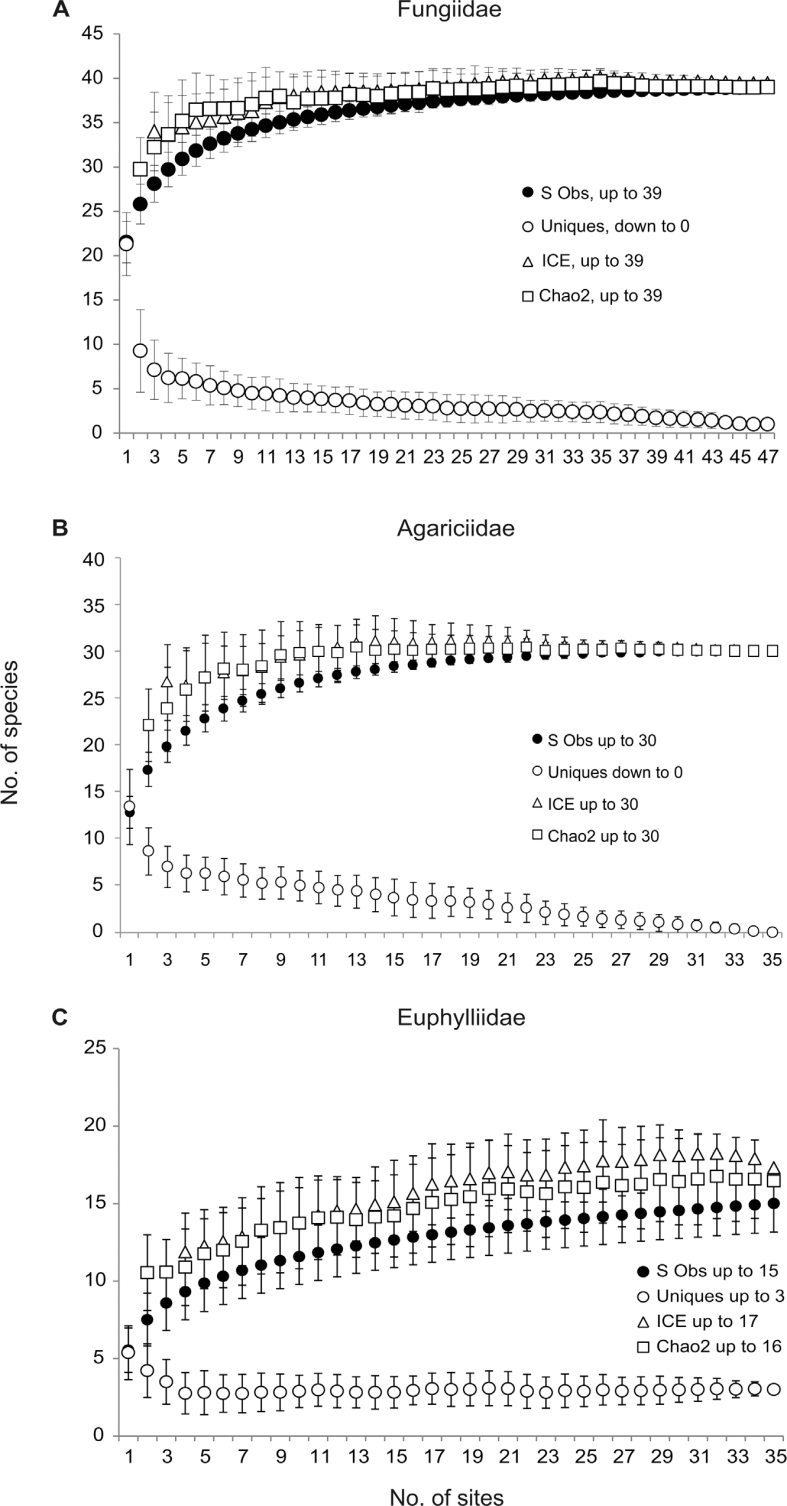
Species richness accumulation curves (observed and estimated). (A) Fungiidae at 47 sites, (B) Agariciidae and (C) Euphylliidae, each at 35 sites.

The species accumulation curves for the fungiid and the agariciid corals showed that the estimated number of species (ICE, Chao 2) is similar to the observed number of species; therefore the sampling has been sufficient (Fig 2). In contrast, the species accumulation curve for the euphylliid corals appears to approach the asymptote, indicating that additional sampling may reveal more species.

Based on the target coral families, the total reef coral species count for the TMP is 84 species, represented by 39 Fungiidae, 30 Agariciidae and 15 Euphylliidae (S3 Table). A total of 18 new records for Kudat (e.g. [29]) include five fungiid species (*Cycloseris boschmai*, *C*. *curvata*, *C*. *distorta*, *Lithophyllon ranjithi*, *Sandalolitha boucheti*), seven agariciids (*Leptoseris fragilis*, *L*. *incrustans*, *L*. *solida*, *Pavona danai*, *P*. *duerdeni*, *Pavona* sp. 1, *Pavona* sp. 2) and six euphylliids (*Euphyllia cristata*, *E*. *paradivisa*, *Nemenzophyllia turbida*, *Plerogyra* cf. *cauliformis*, *Plerogyra* cf. *diabolotus* and *Plerogyra* cf. *multilobata*). In addition to the earlier reported total of 273 scleractinian species for TMP, the 18 new records add up to a new total of 291 species.

The number of coral species observed per site ranged from 13 in Marudu Bay (site 45) to 49 in four separate sites: Purukan Sibaliu (site 15), both sites at the Mangsee Great Reef (sites 26 and 27) and Molleangan Kecil (also known as Maliangin Kecil) at the southern tip of Banggi (site 34). Out of 35 sites, 19 (54%) had a total of ≥ 40 species. The common mushroom coral species *Fungia fungites* was found at all sites, while the mushroom coral *Danafungia fralinae*, and the euphylliid corals *Euphyllia cristata*, *E*. *paradivisa* and *Plerogyra* cf. *diabolotus* were each encountered only once (Table 1).

**Table 1:** The occurrence of common and uncommon coral species in the proposed Tun Mustapha Park.

Common species	Frequency (n)^a^	Uncommon species	Frequency (n ≤ 3)
**Fungiidae**			
*Fungia fungites*	47	*Cycloseris boschmai* ^b^	3
*Danafungia horrida*	46	*Lithophyllon spinifer*	3
*Danafungia scruposa*	46	*Cycloseris curvata*	2
*Herpolitha limax*	46	*Cycloseris distorta*	2
*Lithophyllon repanda*	46	*Sandalolitha boucheti*	2
*Pleuractis granulosa*	46	*Heliofungia fralinae* ^c^	1
*Pleuractis paumotensis*	46		
*Ctenactis echinata*	45		
*Lithophyllon scabra*	45		
*Sandalolitha robusta*	45		
*Lithophyllon concinna*	44		
*Heliofungia actiniformis*	44		
*Cycloseris costulata*	43		
*Pleuractis moluccensis*	43		
*Ctenactis crassa*	43		
**Agariciidae**			
*Pachyseris speciosa*	33	*Leptoseris fragilis*	3
*Pavona explanulata*	31	*Pavona duerdeni*	3
*Pavona varians*	31	*Pavona danai*	2
*Leptoseris scabra*	30	*Pavona* sp. 1	2
		*Pavona* sp. 2	2
**Euphylliidae**			
*Plerogyra sinuosa*	33	*Nemenzophyllia turbida*	3
*Euphyllia ancora*	30	*Plerogyra* cf.*cauliformis*	2
		*Euphyllia cristata*	1
		*Euphyllia paradivisa*	1
		*Plerogyra* cf. *diabolotus*	1

^a^ represented at ≥ 85% of the reef sites: Fungiidae n ≥ 40; Agariciidae, Euphylliidae n ≥ 30.

^b^ from surveys in 2007 and 2008.

^c^ from a survey in 2008 only.

**Fig 3:**
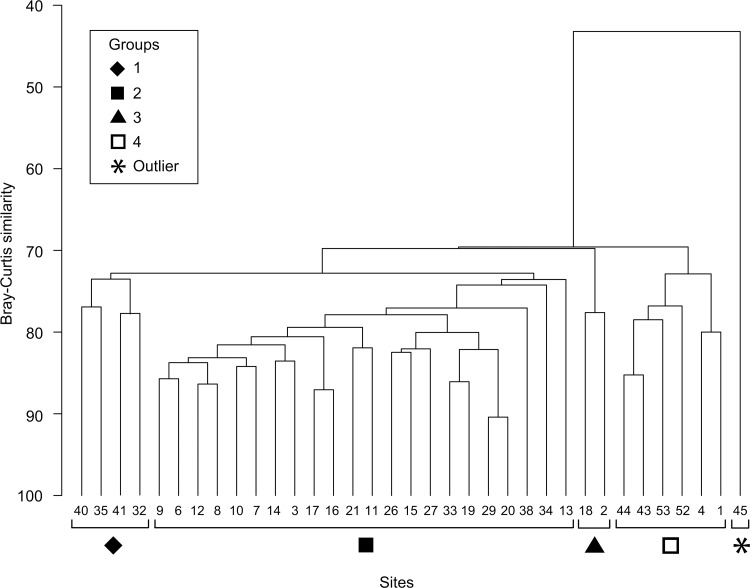
Group-averaged hierarchical clustering dendrogram of coral composition based on the Bray-Curtis similarity index. Coral species composition was based on 35 sites where data was collected for all three coral families. Four significant clusters and one outlier (indicated by the symbols) were computed by the similarity profile (SIMPROF) analysis.

**Fig 4:**
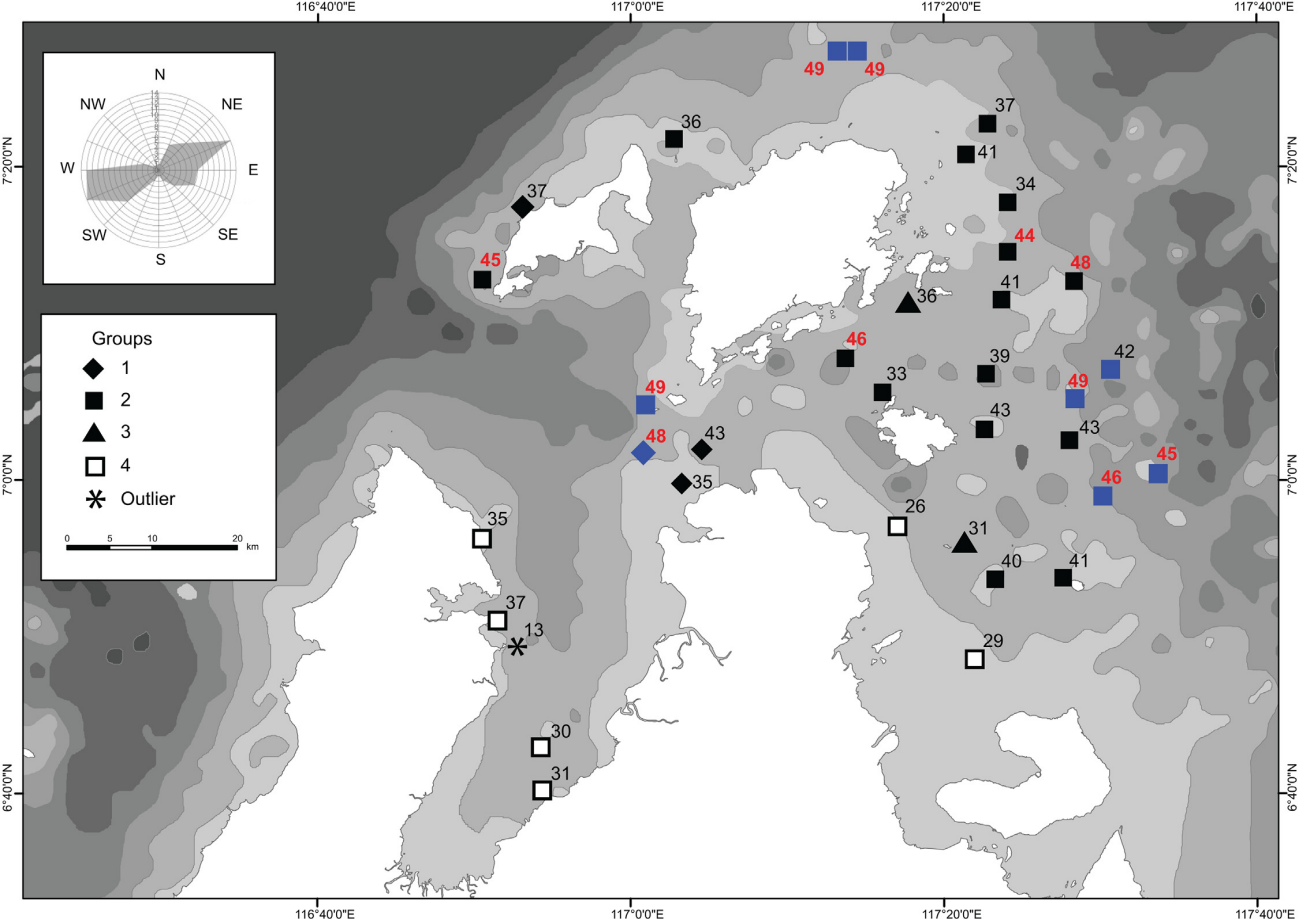
Overlay of the significant clusters from Fig 3 based on the SIMPROF analysis. Symbols in blue represent deep reef sites (> 20 m depth). Numbers indicate the total coral species for all three coral families. Sites with ≥ 45 species are shown in red bold font. Wind rose showing dominant wind directions is based on observations between January 2012 and November 2013 obtained from the Kudat Airport, Sabah from windfinder.com.

The SIMPROF test indicated four distinct clusters and an outlier (site 45) (Fig 3). There was a distinction of two groups at similarity index of ~ 69: the larger of the two groups is further subdivided into two smaller groups (Groups 1 and 3) and one large group (Group 2) at similarity index ~ 70, and another small group (Group 4) that is composed of six sites. The species richness pattern that emerged when the clusters were overlaid on the map showed that sites with high species richness (n ≥ 45) were located primarily at the periphery of the study site (Fig 4). Reef sites that were adjacent to the mainland (Group 4) had noticeably lower species richness (except site 53, n = 38). These sites were among the shallowest sites during the survey.

There was variability in the site conditions, i.e. there were more shallow sites (n = 27) than deep sites (n = 8), and more exposed sites (n = 30) than sheltered sites (n = 5). Approximately 62% of the sites were shallow and exposed. There were no sheltered sites that were deep. The coral species richness appeared to be higher at the deep sites and seemed to increase with distance from the mainland (S2 Fig). Based on the regression analyses, only depth and exposure were significant factors influencing the coral species richness of the reef sites (Table 2, Model 1, S3 Fig). When outlier site 45 was removed from the analyses, exposure became an insignificant factor and only depth had an effect on the species richness in the TMP (Table 2, Model 2). Even though Model 2 had underdispersion in the residuals, its AIC value was smaller and its model diagnostics were better than Model 1, hence Model 2 is a better model based on statistics (S4 Fig).

**Table 2:** The influence of depth, exposure and distance from the mainland on the coral species richness in the TMP.

Model description	Estimate	Std. error	z value	p
**Model 1—**Depth + Exposure + Distance from mainland				
Intercept	3.7899	0.0825	45.9490	< 2e-16
Depth	-0.1852	0.0640	-2.8960	**0.0038**
Exposure	-0.2336	0.1003	-2.3290	**0.0199**
Distance from mainland	0.0019	0.0020	0.9850	0.3248
AIC = 232.76				
Residual deviance = 32.806, on 31 degrees of freedom				
**Model 2**—Depth + Exposure + Distance from mainland (excluding site 45)				
Intercept	3.7920	0.0825	45.9910	< 2e-16
Depth	-0.1858	0.0640	-2.9040	**0.0037**
Exposure	-0.1056	0.1034	-1.0210	0.3072
Distance from mainland	0.0019	0.0020	0.9520	0.3411
AIC = 214.9				
Residual deviance = 19.359, on 30 degrees of freedom				

Significant values are indicated in bold.

### Benthic communities

**Fig 5:**
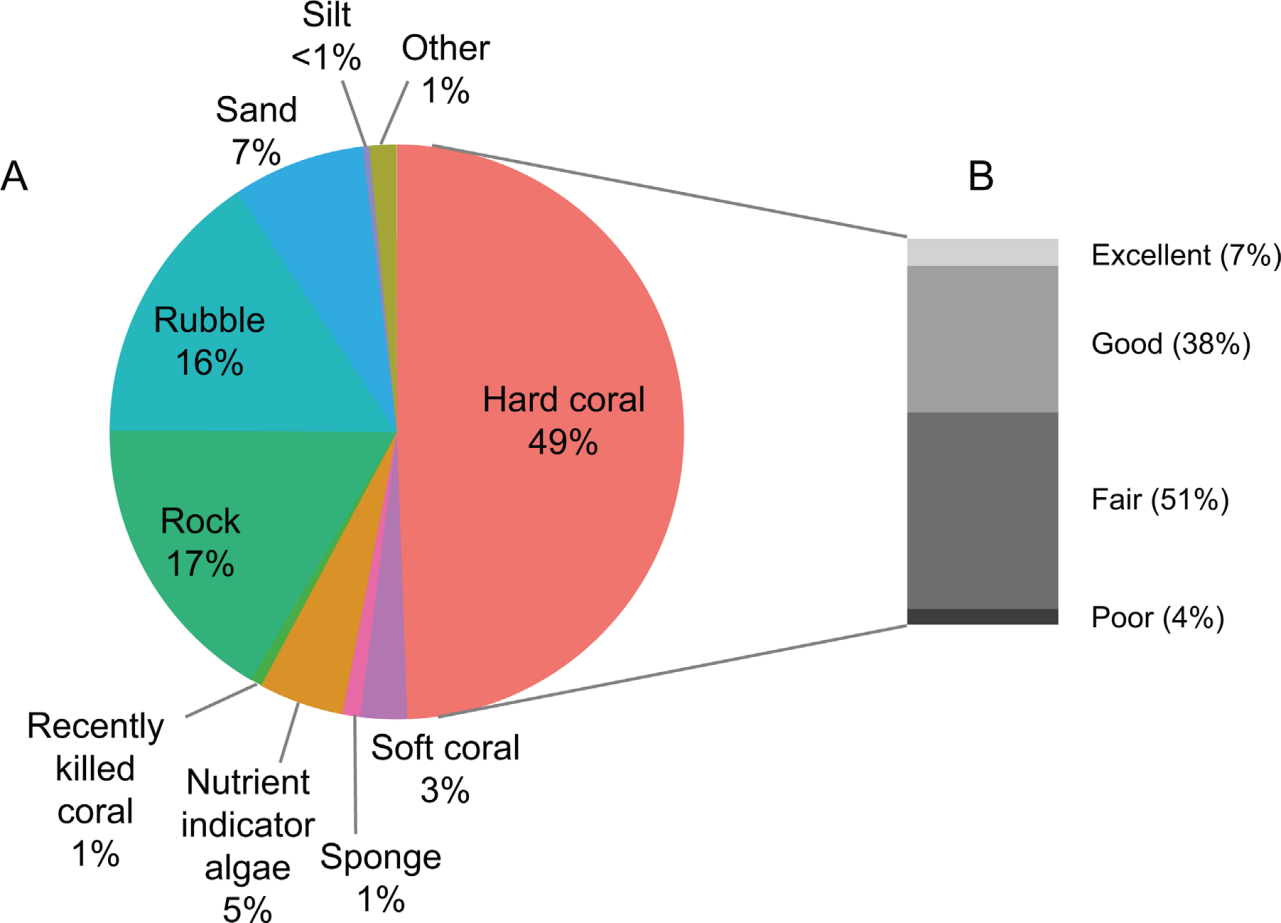
Benthic composition in the proposed Tun Mustapha Park. (A) Average percentage cover of benthic communities, (B) Reef coral status following the criteria of Chou et al. [21].

The average hard coral cover of 55 transects in the TMP was 49% (Fig 5). Following the criteria described by Chou et al. [21], most reefs were rated either fair (51%) or good (38%) in terms of coral cover. Only 7% of the reefs had excellent coral cover, while 4% had poor coral cover. The coral cover ranged from 18% at Pancang Pukul (site 40D) to 77% at Mangsee Reef (site 26S) (S4 Table). Hard coral was the most dominant biotic component on the reef, accounting for almost half of the benthic substrate cover. Other functional biotic components were soft coral (3%, range: 0–31%) and nutrient indicator algae (5%, range: 0–29%). Major abiotic components (> 10% cover) that contributed to the benthic cover were rock (17%, range: 3–34%) and rubble (16%, range: 1–52%).

**Fig 6:**
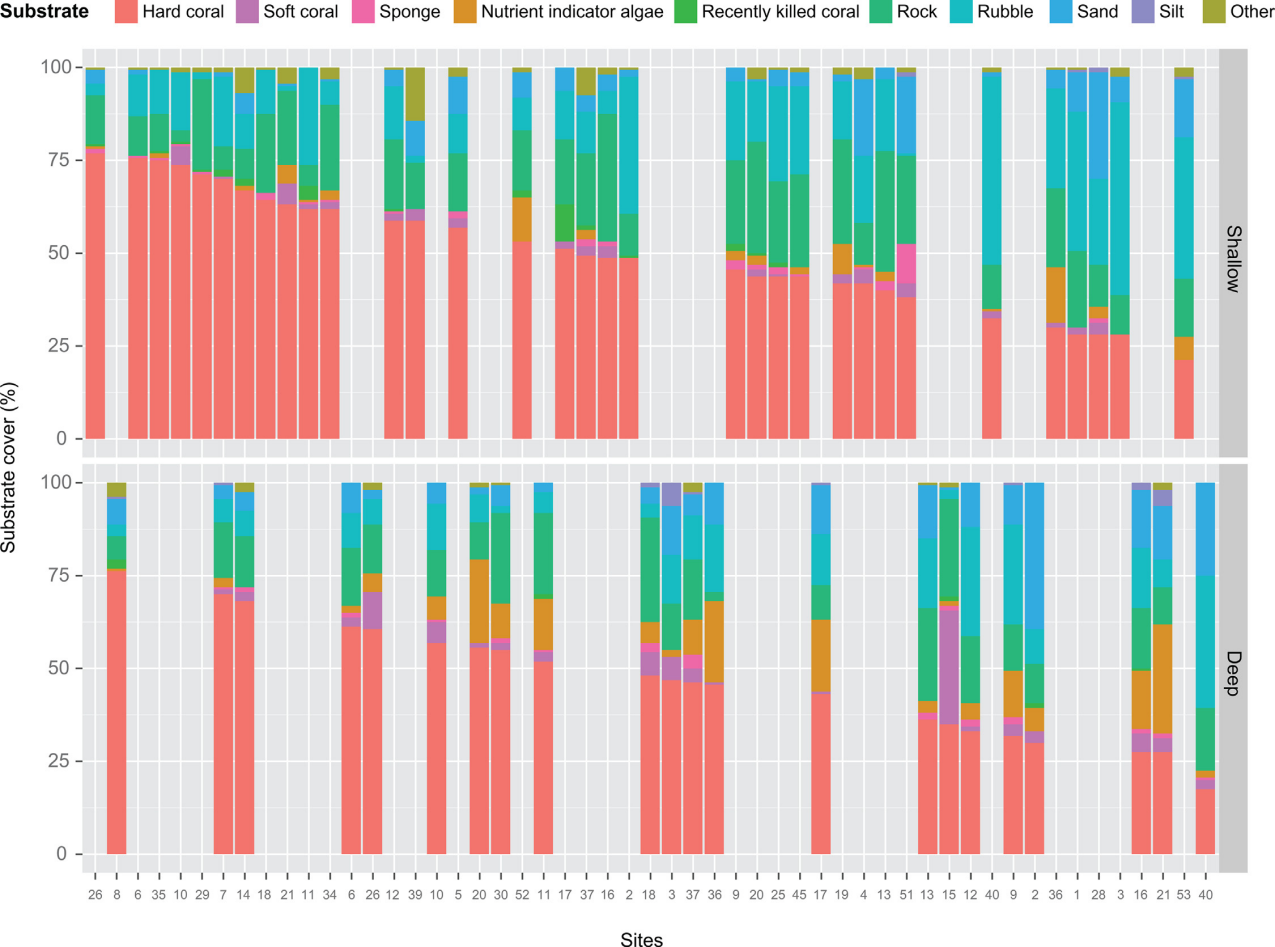
Benthic categories arranged by percentage hard coral cover in decreasing order for shallow and deep transects.

**Fig 7:**
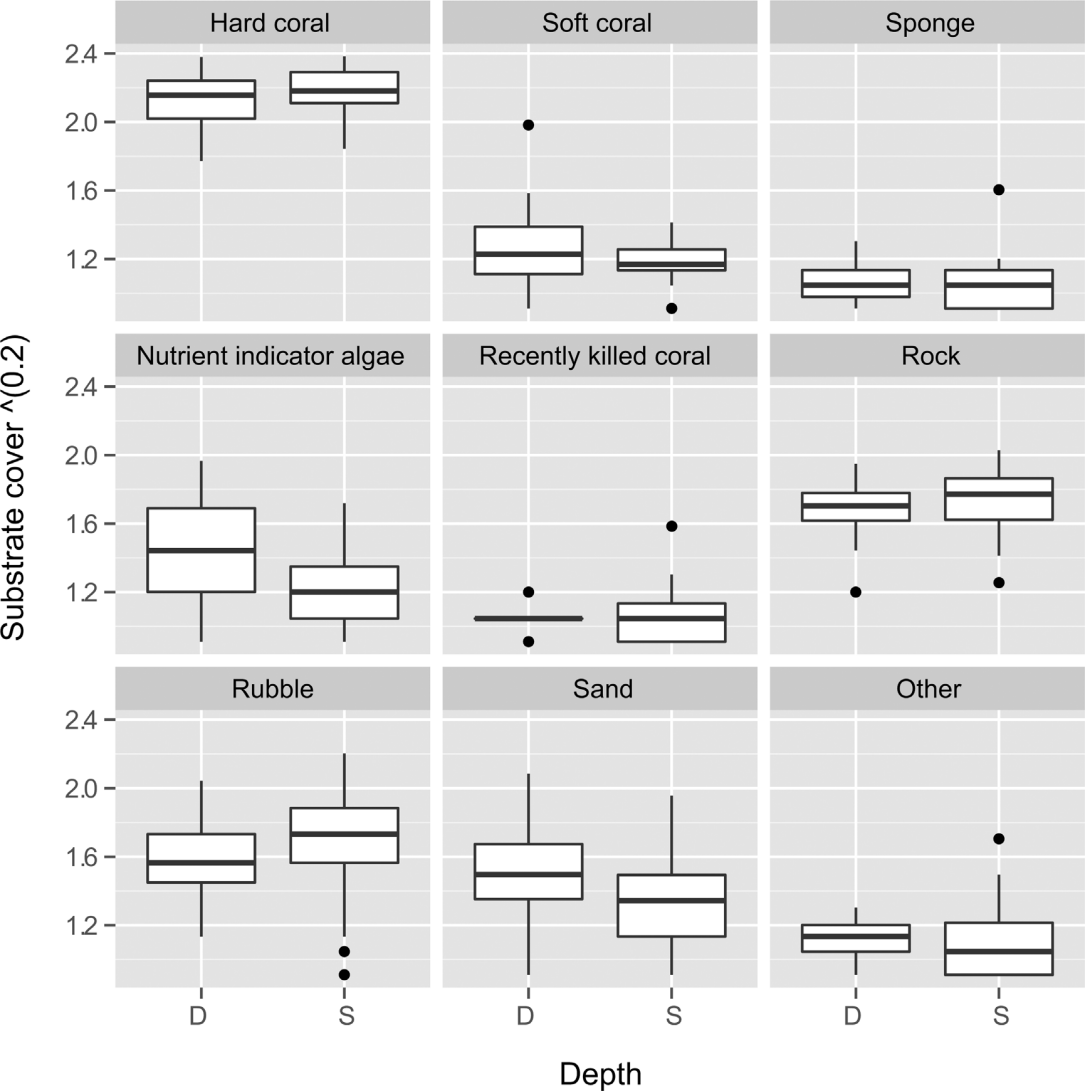
Benthic categories based on shallow and deep transects. Silt was excluded from the analysis as the percentage coverage was very low.

**Fig 8:**
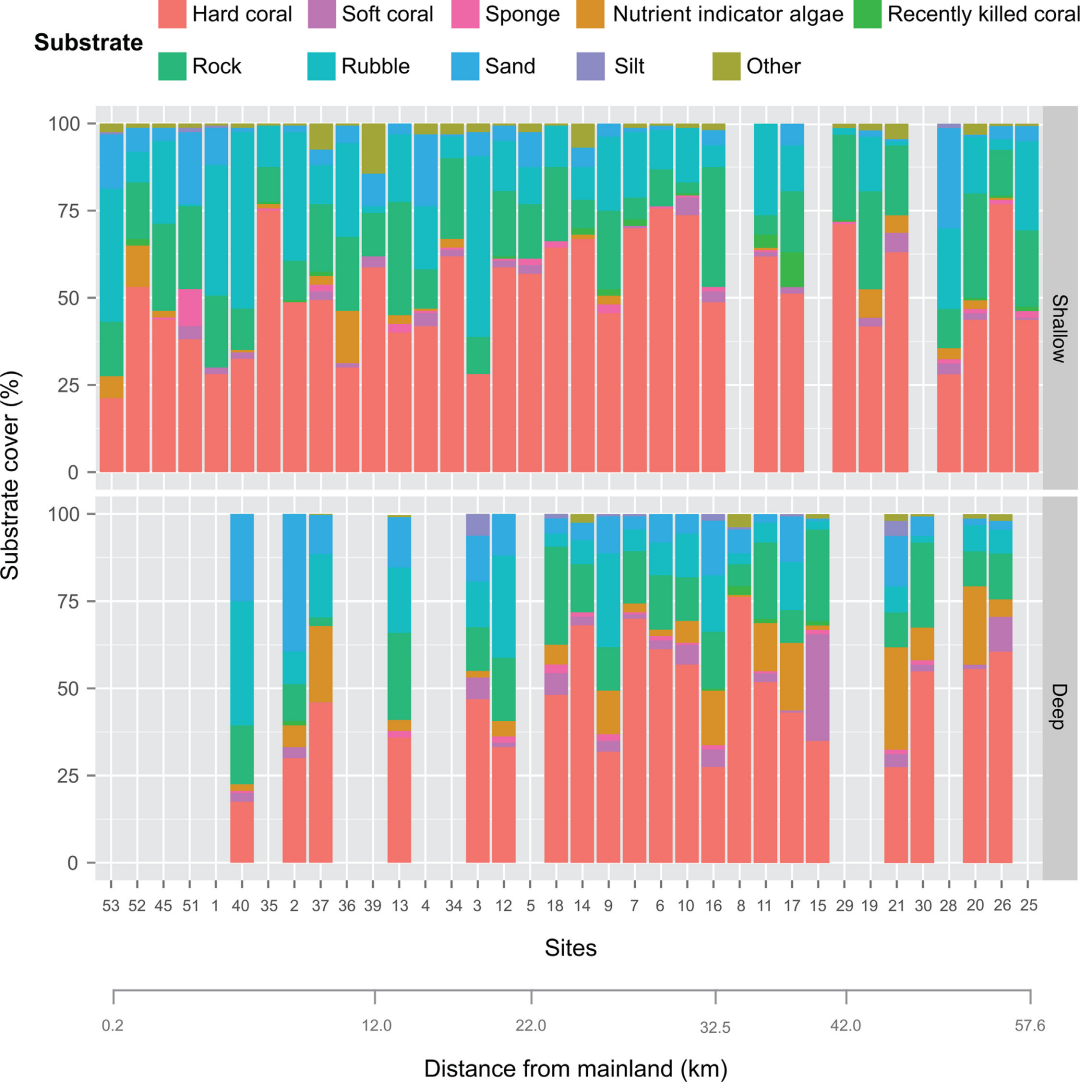
Benthic categories arranged by percentage hard coral cover based on site proximity from the mainland for shallow and deep transects.

As the substrate cover adds up to 100% for each transect, correlations among the substrate categories are negatively related. At sites where hard coral cover was low, rubble was the dominant substrate in the shallow transects, and sand in the deep transects (Figs 6, S5 and S6). In comparing the benthic components between both depths, there was a significant difference in nutrient indicator algae and sand cover, whereby both had higher cover in the deep transects (Figs 7 and S7, Table 3). Nutrient indicator algae was found in 21 deep transects (9%, range: 0–29%) and 17 shallow transects (2%, range: 0–15%), while sand was recorded from all deep transects (12%, range: 1–39%) and 28 shallow transects (6%, range: 0–29%). There seems to be no obvious difference in the structure of the benthic communities when the reef distance from the mainland was examined (Fig 8). Nevertheless, rubble and sand cover decreased with distance from the mainland while hard coral cover increased slightly in both the shallow and deep transects (Table 3, S8 Fig).

**Table 3:** The influence of depth and distance from the mainland on the benthic communities.

Model description	Estimate	Std. error	t value	p
**Depth**				
Hard coral	0.0446	0.0607	0.7350	0.4628
Nutrient indicator algae	-0.2860	0.0933	-3.0660	**0.0023**
Other	-0.0298	0.1040	-2.8700	0.7743
Recently killed coral	-0.0352	0.1290	-0.2730	0.7853
Rock	-0.0012	0.0858	-0.0140	0.9888
Rubble	0.0593	0.0858	0.6910	0.4900
Sand	-0.1910	0.0873	-2.1870	**0.0293**
Soft coral	-0.1333	0.0924	-1.4420	0.1501
Sponge	-0.0534	0.0967	-0.5530	0.5809
Adj. R^2^ = 0.7249				
**Distance from the mainland**				
Hard coral	0.0030	0.0019	1.5720	0.1169
Nutrient indicator algae	-0.0007	0.0029	-0.2370	0.8125
Other	-0.0037	0.0028	-1.3170	0.1886
Recently killed coral	-0.0029	0.0037	-0.7720	0.4403
Rock	-0.0029	0.0027	-1.0630	0.2885
Rubble	-0.0093	0.0027	-3.4650	**0.0006**
Sand	-0.0086	0.0027	-3.1490	**0.0018**
Soft coral	-0.0018	0.0030	-0.5940	0.5528
Sponge	-0.0048	0.0032	-1.5170	0.1300
Adj. R^2^ = 0.7257				

Significant values are indicated in bold.

## Discussion

The new species records bring the total coral count to 291 species in the TMP. This number is higher than the range of 208–262 species found at five of the most diverse Coral Triangle localities in eastern Indonesia, each with nine to 50 dive stations [84]. A recent coral species count at western Sabah, to the southwest of TMP and just outside the Coral Triangle reached a slightly lower maximum of 248 species, taking recent taxonomic revisions into account [30]. Hence, the TMP can be considered rich in coral species and based on the present study an even higher diversity can be expected when other coral families are included.

### Absent, unusual and common coral species

Several species expected to be present based on previous records but not encountered in the present study were the mushroom corals *Heliofungia fralinae* and *Cycloseris boschmai* (from surveys in 2007 and 2008), and the agariciid *Pavona minuta* [29]. The absent mushroom coral *Zoopilus echinatus* was also expected to occur in the area based on its presence in nearby localities in Sabah [48–49] and Brunei [85–86]. All three mushroom coral species are predominantly found in offshore reefs [47, 54, 87–88]. *Pavona minuta* was uncommon and found encrusting on the reef substrate in shallow environments [44], although in oceanic Sipadan Island on the east coast of Sabah, it was found at the opening of a small cave of a steep wall. Its very thin corallum, minute corallites and shallow calicular centres give it a smooth appearance [89]. Small colonies could appear inconspicuous making it easily overlooked on the reef. The absence of these species could imply that TMP lacks offshore reef conditions, or that depth is a limiting factor. In addition, the species accumulation curves for the euphylliids also indicate that more species ought to be found with more sampling effort, especially at sites that were not surveyed at the west coast of the Kudat peninsula, or areas less surveyed such as around Balambangan Island and the offshore Mangsee Reef.

Some coral species exhibit explicit habitat preference and appear to be limited to a certain environment based on the reef type or depth [47, 54, 90]. Among the corals that are habitat specialist and uncommon in our study were the fungiids *Cycloseris curvata*, *C*. *distorta* and *Sandalolitha boucheti*, and the agariciids *Pavona duerdeni* and *Leptoseris fragilis*. These rare species, like *Heliofungia fralinae* and *Cycloseris boschmai*, are usually found in offshore reef habitats with clear waters [47–48, 50, 91–93]. *Leptoseris* corals are also known to be deep-reef dwellers and are important components of mesophotic reefs [94–96]. Among species associated with shallow reefs, i.e. *Pavona danai*, *Euphyllia cristata* and *E*. *paradivisa*, the former two species were found near the Bankawan reef flat. Species with a preference for sheltered and occasional turbid conditions are *Lithophyllon spinifer*, *Nemenzophyllia turbida*, *Plerogyra* cf. *cauliformis* and *Plerogyra* cf. *diabolotus* [48, 74]. Coral species with known habitat preference can be useful indicators of environmental conditions in an area. Other endemic species such as the euphylliids *Plerogyra cauliformis*, *and P*. *diabolotus* have only been found in Semporna thus far [48, 74]. In Kudat, specimens that resembled these species and *P*. *multilobata* were not typically as described by Ditlev [74], especially in their characteristic vesicle appearance. However, specimens of these species were not typical of the other closest species either, i.e. *Plerogyra simplex* (for *P*. *cauliformis*) and *Plerogyra sinuosa* (for *P*. *diabolotus* and *P*. *multilobata*).

There were two species of *Pavona* corals that were found in Kudat that do not fit the description of extant species from the Indo-Pacific. *Pavona* sp. 1 (labeled *Pavona* sp. by Waheed and Hoeksema [49]) superficially resembles *Pavona diffluens* (see [44]), only known from the Red Sea, the Arabian Sea and the Persian Gulf. Only two specimens were found and both were from the sediment-laden waters of Marudu Bay (sites 52 and 53). There is a possibility that *Pavona* sp. 1 represents an ecophenotypic variation of *Pavona explanulata*, the closest resembling species, which has adapted to shallow, low-light and sandy substratum condition. Two specimens of *Pavona* sp. 2 (sites 3 and 10) have encrusting growth-form and appear to have very coarse corallites (S9 Fig).

Common corals in the TMP are species that have widespread distributions and are ordinarily found on most western Indo-Pacific reef habitats (listed in Table 1). The mushroom coral *Fungia fungites* was the only species present at all sites. This species is usually common in shallow depths [46–47, 67], where it can reproduce asexually by budding [97]. Other common coral species here have also been reported as dominant corals in the reefs of Sabah [48–49] and other parts of the Indo-Pacific [44].

### Coral species richness patterns

Highest coral species richness is commonly found on mid-shelf reefs [51–54, 58] but the reefs within the TMP did not exhibit a clear cross-shelf zonation pattern as most of the reefs are along gentle slopes across the study area before descending into greater depths at the border of the proposed park. The highest coral species richness was found on the reefs at the peripheral area of the study site away from the mainland (n = 8), and at reefs situated near the South Banggi Channel (n = 3). These reefs were among the deepest sites in the study area and exposed to strong currents that are most likely driving the coral composition on the reefs (e.g. [55]). The deep reefs of the TMP also had a shallow reef zone, and this wider depth range could accommodate coral species with a preference for shallow as well as deep environments, therefore adding species richness to these reefs. Exposure was not significant in influencing the species richness patterns, but this could be due to the high variance in the dataset, as there were very few sites in the sheltered reef condition (approximately 15% of the surveyed reef area only). In comparison with reef studies elsewhere in Sabah, an almost similar pattern was observed for the reefs of Kota Kinabalu, whereby the deeper (and exposed) reefs had higher species richness [49]. In contrast to the reefs of Semporna, the highest species richness was noted on the sheltered nearshore reefs [48].

Perceptibly lower species richness was seen at the reefs fringing the mainland, as indicated by the outlier site 45 and cluster of Group 4. For the latter, all six sites were shallow, of which only four sites were sheltered from the dominant winds, while two other sites were exposed reefs fringing the west coast of the Bengkoka peninsula, suggesting that this cluster was grouped based on its proximity to the mainland. These fringing reefs tend to be in turbid waters and the coral species here have presumably adapted to low light condition. Wood [17] attributed the characteristics of the reef fauna and the lack of vertical reef development in the coastal reefs to the shallow depths and close proximity to shore. Although the proximity to the mainland was not as significant as depth in influencing the species richness patterns in the TMP, a difference in species composition could be discerned, as was also the case for reefs in Indonesia [54–55].

Approximately 85% of the surveyed reefs in the TMP were considered exposed reefs but they appear to be compositionally different in terms of coral species and hence clustered further into three groups (Fig 3). Group 1 had three sites in the South Banggi Channel and one site at the west of Balambangan Island, which are characterized by very strong currents. The west coast of Balambangan had a different reefscape from the rest of the sites in the TMP by means of low-lying reefs with little profile development. These reef have been described as having a cyclic die-off and regrowth of alga-consolidated reefs [15] caused by repetitive sediment-resuspension from strong current and wave force. Despite the lack in reef development, these reefs have been shown to have high coral diversity, as was also reported in other areas with non-reef-building coral environments [98].

### Reef benthos assemblages

Hard coral cover in the TMP (49%) exceeded the average values of other areas in the Indo-Pacific. Surveys in 2003 indicated that coral cover from 390 reefs in the region averaged only 22% [99]. Looking at reefs closer to the TMP, the average coral cover in Tubbataha Reef, the Philippines some 320 km away was 36% in the deep sites and 40% in the shallow sites [100]. Elsewhere in Sabah, the average percentage cover was 41% in Lankayan Island, Sandakan [101] and 37% in Semporna [102]. The methods for collecting hard coral cover data were not necessarily the same, but were sufficiently similar so as to enable a general comparison across the region.

Some earlier data on the reef status of the TMP is available for comparison, but cannot be used as baseline to measure changes in coral cover as different sites were surveyed. The Pulau Banggi Project for Coral Reef Biodiversity established permanent monitoring sites in the southeast of Banggi with a sampling series of three years from 1999 to 2002. There was no significant difference in mean coral cover in four permanent sites between the first two years [19]. Other surveys were conducted at different reef sites in order to fill the information gap in the area. The findings from these surveys between 2002–2004 and 2009 showed that only 5% of the reefs had excellent coral cover and 9% had poor coral cover, while most were good (31%) or fair (55%) [22–25]. This is comparable with our results (reefs with 7% excellent, 38% good, 51% fair and 4% poor coral cover).

The comparison between the shallow and deep transects revealed a significant difference in the cover of nutrient indicator algae. Algae are commonly known to correspond with areas of high nutrient input or low grazers or herbivores [103–105]. Input of nutrients is usually a concern for reefs near urban areas [103], but our research site is considered remote with no major urban development nearby. Furthermore, proximity to the mainland had no effect on the algae cover (Fig 8, Table 3). In previous surveys, algae cover was highly variable (0–49%) [22–24], but was usually more abundant in shallow depths [18–19]. This is in contrast with our findings where algae cover was higher in the deep transect. Higher than average algal cover (between 13%–29%) at some sites has been attributed to possible nutrient input from river mouths, [22] or shoreline vegetation [23].

Sand cover was found to be high at the deep transects and decreased with distance from the mainland, which is consistent with the reef geomorphology of the TMP area. The fringing reefs are generally in shallow and turbid environments. It was common to find large stretches of sand among the patch reefs and reef flats. Moving further away from the mainland, most shallow reefs show a gentle incline before continuing into sandy substrate between 15–20 m depths.

Rubble cover was previously reported to be higher in the shallow reef sites (see [19, 24–25]). A similar observation was made in the present study with an additional note that rubble cover appears to decrease with distance from the mainland. It is difficult to distinguish the main cause of high rubble cover as the differences between the effects from blast fishing and storm damage are subtle and can appear visually similar, especially in shallow reefs and areas where blast damage is old. The fact that blast fishing has been occurring in the area since the 1970s [106] and has been reported to be the primary threat to the reefs suggests that the presence of rubble in these reefs is most likely caused by this practice [107–108]. It is likely that blast fishing mostly occur at reefs closer to inhabited islands as it would take more effort to reach the more remote reefs, however, data is unavailable as evidence. Previously reported blast frequency was 0.8–1.3 incidences/hour in the Banggi region [18, 20]. Several reef sites had unconsolidated coral fragments as evidence of previous blast fishing (e.g. sites 3S and 10S), but the observed damage on the reef appeared old. Damage from blast fishing may have a long-lasting effect, especially when it occurs at a large scale and over a prolonged period of time [107–109]. Throughout the survey 15 blasts were heard with a maximum of six detonations in one dive. Signs of recent blast impact were also witnessed in the form of dead (and dying) fish at Kalutan Island (site 33) that most likely drifted there with the currents as the reef was intact and had one of the highest coral species richness in the TMP. Storm damage was mostly observed at the exposed reef flats of Bankawan and Carrington Reef (pers. obs).

Although the Reef Check method is not able to indicate some ecological measures, such as the quality of the reefs, it is able to give snapshots of the reef status, hence shedding light on the reef condition of which very little is known of in the TMP. As a note, a reef with high coral cover does not give any indication to the health of the reef (see [110]). Nevertheless, an advantage of the Reef Check protocol is that its standard monitoring categories allows for comparison of data across large areas [75] in order to know the status of a particular reef in relation to other reefs. This is a useful start for the TMP where not much data is available from the area and monitoring protocols are not yet in place.

## Conclusions

Plans are underway for the establishment of the Tun Mustapha Park by the end of 2015 and the park management will adopt a multiple-use concept, whereby various zones will be identified for different uses, while at the same time ensuring conservation and sustainable use of natural resources [111–112]. The present study shows that reefs with high species richness are found at the periphery of the TMP boundary away from the mainland and near the South Banggi Channel. Most of these reefs extend to deeper depths, as compared to the rest of the reefs within the proposed park. Depth is an important factor in influencing the coral species composition and some aspects of the benthic reef assemblages. The proximity to the mainland, though not significant as depth, also plays a part in structuring the coral species richness. Furthermore, reefs with good and poor coral cover have been identified. When these layers of information are overlaid on the TMP map, particular reefs stand out as being important from a conservation perspective and thus should be afforded protection under the preservation zones. A continuous monitoring programme should be established by setting up permanent monitoring sites within the TMP. Information collected from these sites would be crucial to evaluate temporal changes or assess impacts on the coral reefs [11].

## Supporting Information

S1 Fig Additional sites surveyed for only Fungiidae between 2005 and 2008. (PDF)

S2 Fig Visual analysis. Exploring the coral species richness data.
(PDF)

S3 Fig Model 1. Full model with all three environmental factors depth, exposure and distance to the mainland.
(PDF)

S4 Fig Model 2. Model 1 excluding outlier site 45.
(PDF)

S5 Fig Visual analysis. Exploring the benthic community data across all transects in accordance to the distance from the mainland.
(PDF)

S6 Fig Correlation between hard corals and other dominant substrate components (rubble and sand). (PDF)

S7 Fig Model 3. Examining the effect of depth on the benthic communities.
(PDF)

S8 Fig Model 4. Examining the effect of distance from the mainland on the benthic communities.
(PDF)

S9 Fig *Pavona* spp. (A) *Pavona* sp. 1 from site 53, (B) *Pavona* sp. 2 from site 3. (PDF)

S1 Table Locality data of coral species diversity and benthic surveys in the proposed Tun Mustapha Park. The distance between the sites for both surveys ranged from 0.16 to 5.80 km apart of each other.
(XLSX)

S2 Table Depth, exposure and distance from the mainland of sites for the coral diversity and benthic community surveys. (XLSX)

S3 Table Fungiidae, Agariciidae and Euphylliidae species incidence. For Fungiidae, observations were made at 38 sites during the Tun Mustapha Park Expedition (TMPE) 2012 and nine sites from previous surveys in 2005, 2007 and 2008. For Agariciidae and Euphylliidae, observations were made at 35 sites during the Tun Mustapha Park Expedition (TMPE) 2012.
(XLSX)

S4 Table Reef substrate composition based on the benthic surveys in the proposed Tun Mustapha Park. Data was collected from 33 shallow and 22 deep reef sites.
(XLSX)

S5 Table Dataset for coral species richness analyses. (TXT)

S6 Table Dataset for benthic community analyses. (TXT)
