# Supplementary material for: Coral Reefs at the Northernmost Tip of Borneo: An Assessment of Scleractinian Species Richness Patterns and Benthic Reef Assemblages
Source: PLoS One. 2015 Dec 31;10(12):e0146006. doi: 10.1371/journal.pone.0146006 (PMC4697805; doi:10.1371/journal.pone.0146006)
Supplement: S1 Fig — (PDF) [file pone.0146006.s001.pdf]

S1 Fig. Additional sites surveyed for only Fungiidae between 2005 and 2008.

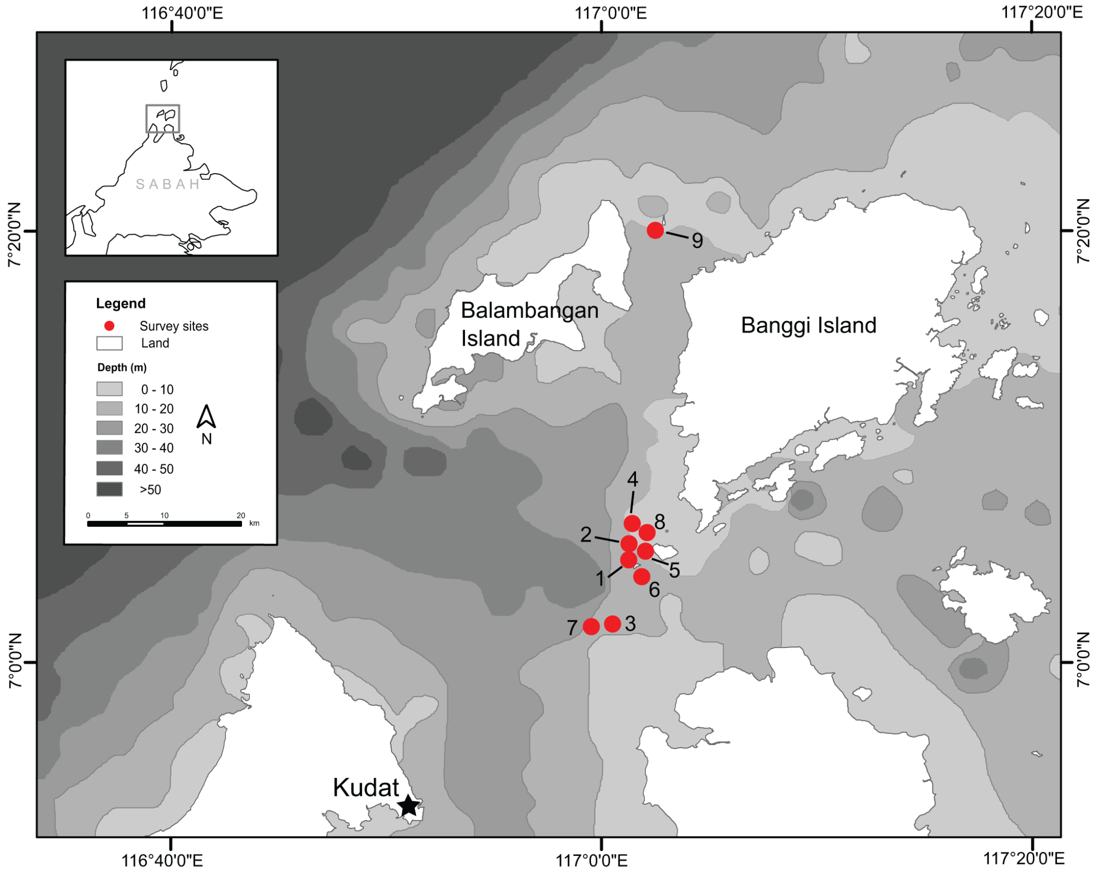

Date	Site no.	Site name	Latitude (N)	Longitude (E)
30 July 2005	1	Molleangan Kecil Is., NW	7°04'56.0"	117°01'03.0"
30 July 2005	2	Molleangan Besar Is., W	7°05'20.0"	117°01'15.0"
8 August 2007	3	Belaruan	7°02'08.0"	117°00'37.0"
8 August 2007	4	Molleangan Besar Is., NW	7°05'39.0"	117°01'30.0"
19 Sept 2008	5	Molleangan Besar Is., W	7°05'16.0"	117°01'31.0"
19 Sept 2008	6	Molleangan Kecil Is., SE	7°04'27.0"	117°01'38.0"
19 Sept 2008	7	Belaruan	7°02'07.0"	117°00'06.0"
20 Sept 2008	8	Molleangan Besar Is., NW	7°05'34.0"	117°01'34.0"
20 Sept 2008	9	Tiga Is.	7°20'16.0"	117°02'35.0"
