# Supplementary material for: Coral Reefs at the Northernmost Tip of Borneo: An Assessment of Scleractinian Species Richness Patterns and Benthic Reef Assemblages
Source: PLoS One. 2015 Dec 31;10(12):e0146006. doi: 10.1371/journal.pone.0146006 (PMC4697805; doi:10.1371/journal.pone.0146006)
Supplement: S2 Fig — Exploring the coral species richness data. (PDF) [file pone.0146006.s002.pdf]

S2 Fig. Visual analysis. Exploring the coral species richness data.

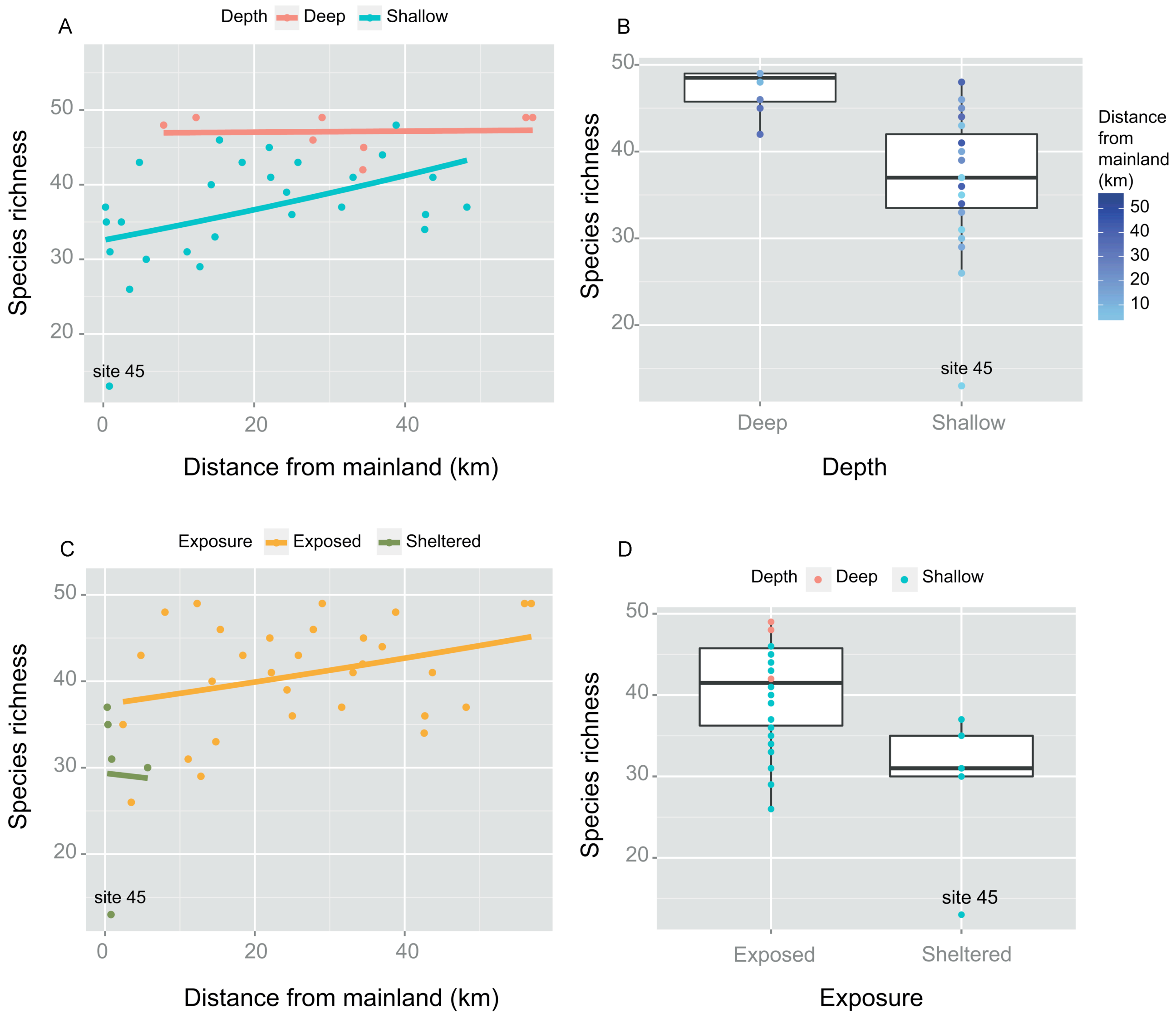

A. Species richness seems to increase with distance from the mainland in the shallow reefs. The deep reefs were not affected by the distance from the mainland. Site 45 is species-poor.

B. Species richness is higher at the deep reef sites, but there are fewer survey sites in the deep reefs. There is no clear structure that species richness increases with distance from the mainland (contrary to the shallow reefs as seen in A).

C. All sheltered reefs were less than 10 km from the mainland. Sheltered reefs were not as species rich as the exposed reefs.

D. Species richness is higher in exposed reef sites, but there are more sites in the exposed reefs than sheltered reef conditions. All deeps reefs were in exposed conditions.
