# Supplementary material for: Coral Reefs at the Northernmost Tip of Borneo: An Assessment of Scleractinian Species Richness Patterns and Benthic Reef Assemblages
Source: PLoS One. 2015 Dec 31;10(12):e0146006. doi: 10.1371/journal.pone.0146006 (PMC4697805; doi:10.1371/journal.pone.0146006)
Supplement: S3 Fig — Full model with all three environmental factors depth, exposure and distance to the mainland. (PDF) [file pone.0146006.s003.pdf]

S3 Fig. Model 1. Full model with all three environmental factors depth, exposure and distance to the mainland.

GLM was used to analyse the model for the species richness data (count data). ANOVA type II was used as it is better suited for unbalanced design (fewer observations in certain variables).

```
modelGLM<-glm(Richness~Depth+Exposure+Dist.mainland, family=poisson, data=Richness)
##
library(car)
##
Anova(modelGLM)
##
## Analysis of Deviance Table (Type II tests)
##
## Response: Richness
##               LR Chisq Df Pr(>Chisq)
## Depth           8.1969  1   0.004196 **
## Exposure        5.5933  1   0.018029 *
## Dist.mainland   0.9685  1   0.325043
## ---
## Signif. codes:  0 '***' 0.001 '**' 0.01 '*' 0.05 '.' 0.1 ' ' 1
##
## summary(modelGLM)
##
## Call:
## glm(formula = Richness ~ Depth + Exposure + Dist.mainland, family = poisson,
##     data = Richness)
##
## Deviance Residuals:
##     Min       1Q   Median       3Q      Max
## -3.3629  -0.4779   0.1047   0.6143   1.3998
##
## Coefficients:
##                    Estimate Std. Error z value Pr(>|z|)
## (Intercept)        3.789882   0.082480  45.949  < 2e-16 ***
## DepthShallow      -0.185239   0.063972  -2.896  0.00378 **
## ExposureSheltered -0.233607   0.100308  -2.329  0.01986 **
## Dist.mainland      0.001928   0.001959   0.985  0.32481
## ---
## Signif. codes:  0 '***' 0.001 '**' 0.01 '*' 0.05 '.' 0.1 ' ' 1
##
## (Dispersion parameter for poisson family taken to be 1)
##
##     Null deviance: 60.051  on 34  degrees of freedom
## Residual deviance: 32.806  on 31  degrees of freedom
## AIC: 232.76
##
## Number of Fisher Scoring iterations: 4
```

ANOVA and the summary table shows that covariate distance from the mainland is not a significant factor. The model has slight overdispersion (residual deviance = 32.806 on df 31) indicating a good fit.

Model diagnostics

```r
qqPlot(modelGLM$residuals)
residualPlots(modelGLM)
```

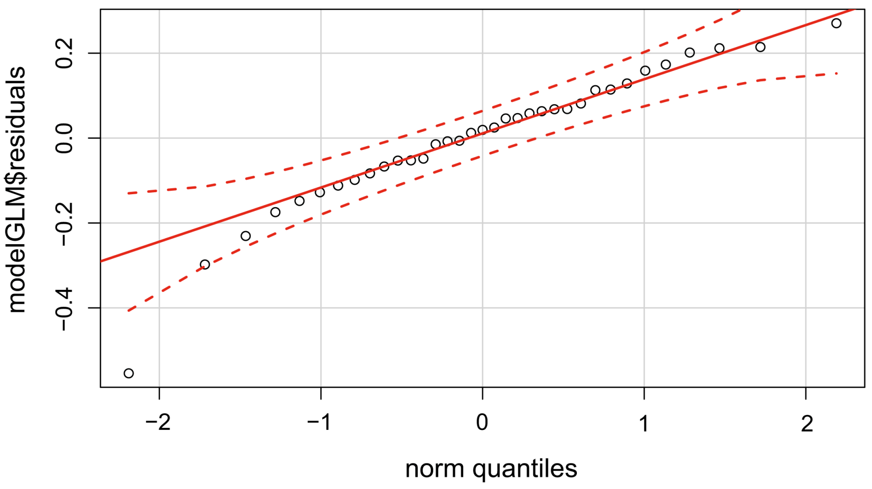

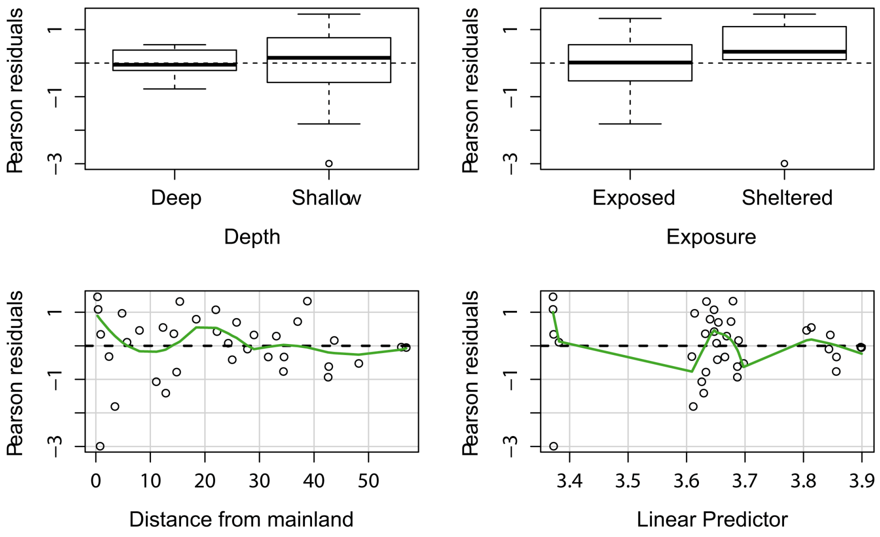


```
##                Test stat Pr(>|t|)
## Exposure              NA       NA
## Depth                 NA       NA
## Dist.mainland      0.753    0.386
```

The transformed residuals are distributed evenly and did not deviate much from the normality assumption. In the Pearson residuals vs. exposure marginal plot, site 45 (outlier) has some negative influence on the model. The other diagnostics are in line with the assumptions and no non-linearity is indicated in the relation between richness and distance from the mainland.

```r
influenceIndexPlot(modelGLM, vars= c("Cook", "hat"))
```

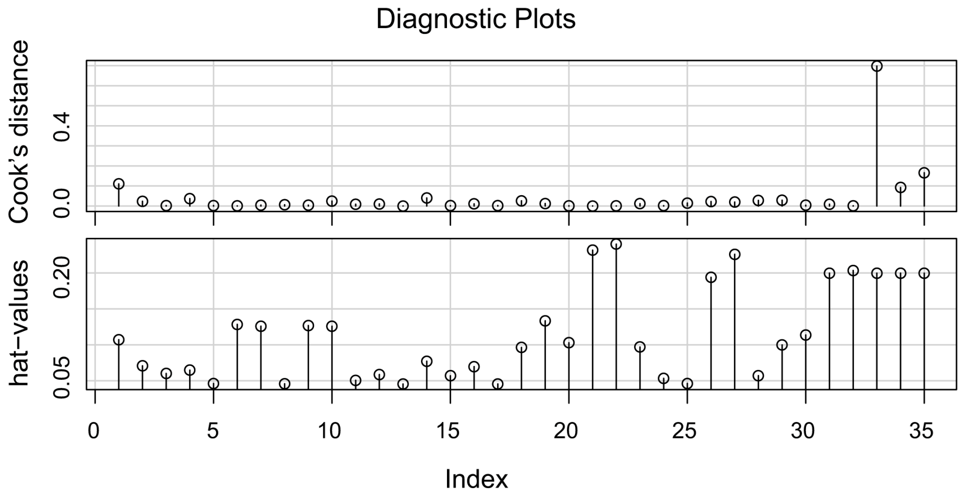


```r
outlierTest(modelGLM)
##      rstudent unadjusted p-value Bonferonni p
## 33  -3.680101         0.00023314      0.00816

Richness$Site[33]
## [1] 45
```

Observation 33 (site 45) is an outlier.

The plots indicate rather good diagnostics. Only observation 33 (site 45) is identified with a high Cook's distance (0.7) and observation 14 (site 15) has a large hat value (influential point).
