# Supplementary material for: Coral Reefs at the Northernmost Tip of Borneo: An Assessment of Scleractinian Species Richness Patterns and Benthic Reef Assemblages
Source: PLoS One. 2015 Dec 31;10(12):e0146006. doi: 10.1371/journal.pone.0146006 (PMC4697805; doi:10.1371/journal.pone.0146006)
Supplement: S4 Fig — Model 1 excluding outlier site 45. (PDF) [file pone.0146006.s004.pdf]

S4 Fig. Model 2. Model 1 excluding outlier site 45.

Analysis was repeated with excluding outlier site 45 from the model.

```
modelGLM2<-glm(Richness~Depth+Exposure+Dist.mainland, family=poisson,
data=Richness[-33,])

Anova(modelGLM2)
## Analysis of Deviance Table (Type II tests)
##
## Response: Richness
##                LR Chisq Df  Pr(>Chisq)
## Depth            8.2439  1    0.004089 **
## Exposure         1.0589  1    0.303475
## Dist.mainland    0.9054  1    0.341349
## ---
## Signif. codes:  0 '***' 0.001 '**' 0.01 '*' 0.05 '.' 0.1 ' ' 1
##
## summary(modelGLM2)
##
## Call:
## glm(formula = Richness ~ Depth + Exposure + Dist.mainland, family = poisson,
##     data = Richness[-33, ])
##
## Deviance Residuals:
##      Min        1Q    Median        3Q       Max
## -1.92190  -0.49932   0.02148   0.50879   1.29314
##
## Coefficients:
##                     Estimate   Std. Error z value Pr(>|z|)
## (Intercept)         3.791990     0.082450  45.991  < 2e-16 ***
## DepthShallow       -0.185767     0.063969  -2.904  0.00368 **
## ExposureSheltered -0.105580     0.103405  -1.021  0.30724
## Dist.mainland       0.001864     0.001958   0.952  0.34112
## ---
## Signif. codes:  0 '***' 0.001 '**' 0.01 '*' 0.05 '.' 0.1 ' ' 1
##
## (Dispersion parameter for poisson family taken to be 1)
##
##     Null deviance: 35.875  on 33  degrees of freedom
## Residual deviance: 19.359  on 30  degrees of freedom
## AIC: 214.9
##
## Number of Fisher Scoring iterations: 4
```

Excluding site 45 resulted in underdispersion in the model (residual deviance= 19.359 on 30 df). A quasi poisson does not change the interpretation of the statistics. The AIC value without site 45 has decreased compared with the model including the outlier.

Due to the very few sites under sheltered reef conditions, and that site 45 is an outlier, we cannot make any well found decision on the effect of exposure on the coral species richness.

Model diagnostics

```r
qqPlot(modelGLM2$residuals)
residualPlots(modelGLM2)
```

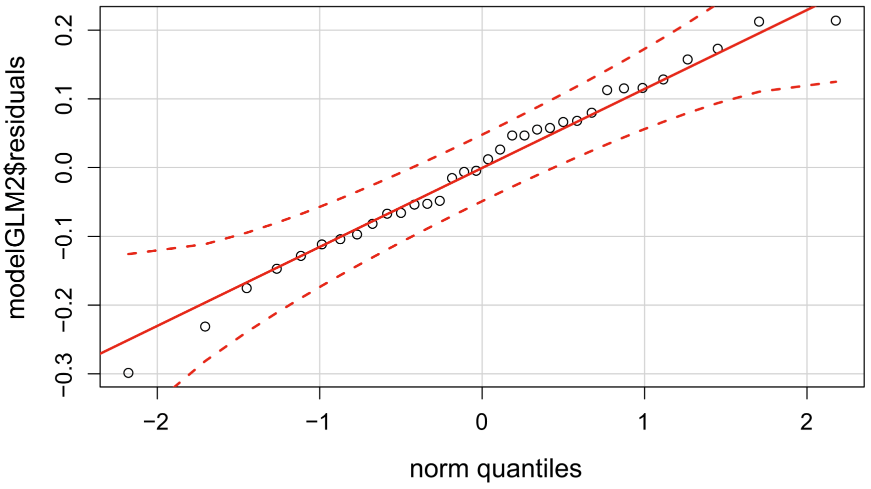

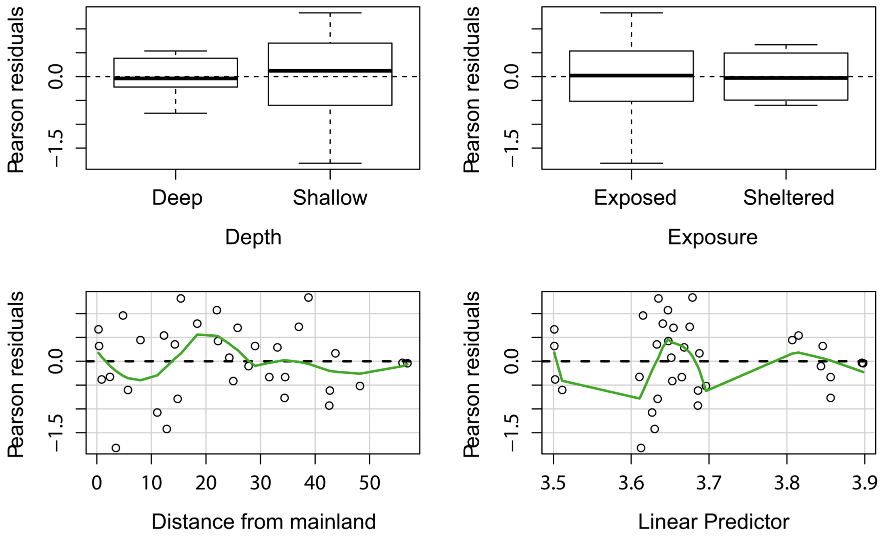


```
##                Test stat Pr(>|t|)
## Depth                 NA       NA
## Exposure              NA       NA
## Dist.mainland      0.591    0.442
```

```r
influenceIndexPlot(modelGLM2, vars= c("Cook", "hat"))
```

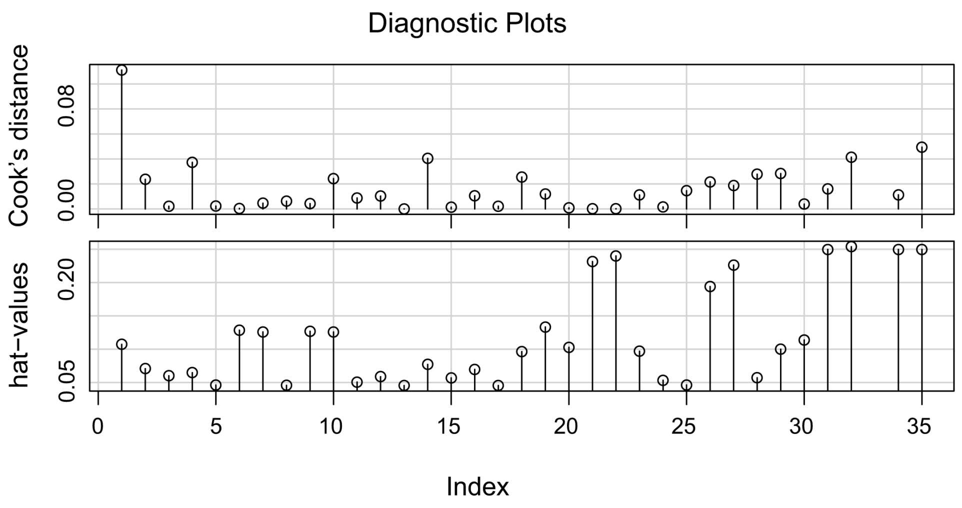

The model diagnostics look better than that of Model 1.
