# Supplementary material for: Coral Reefs at the Northernmost Tip of Borneo: An Assessment of Scleractinian Species Richness Patterns and Benthic Reef Assemblages
Source: PLoS One. 2015 Dec 31;10(12):e0146006. doi: 10.1371/journal.pone.0146006 (PMC4697805; doi:10.1371/journal.pone.0146006)
Supplement: S5 Fig — Exploring the benthic community data across all transects in accordance to the distance from the mainland. (PDF) [file pone.0146006.s005.pdf]

S5 Fig. Visual analysis. Exploring the benthic community data across all transects in accordance to the distance from the mainland

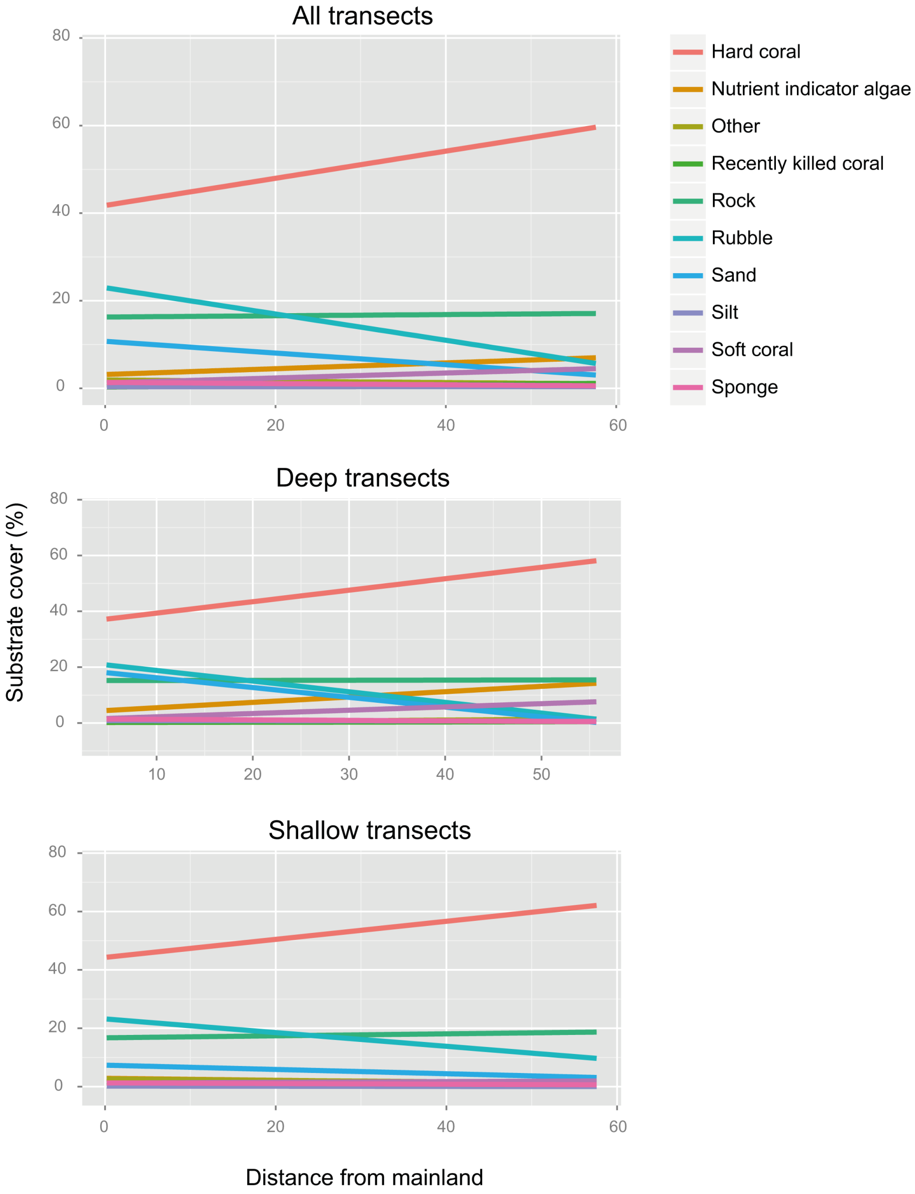

These graphs are similar to Fig. 6 except a smoother function was applied to the data (to create regression lines through each benthic categories) in order to explore the overall trend of the benthic communities in relation to the distance from the mainland. Hard coral seems to increase, while rubble and sand appear to decrease with distance from the mainland.
