# Supplementary material for: Coral Reefs at the Northernmost Tip of Borneo: An Assessment of Scleractinian Species Richness Patterns and Benthic Reef Assemblages
Source: PLoS One. 2015 Dec 31;10(12):e0146006. doi: 10.1371/journal.pone.0146006 (PMC4697805; doi:10.1371/journal.pone.0146006)
Supplement: S6 Fig — (PDF) [file pone.0146006.s006.pdf]

S6 Fig. Correlation between hard corals and other dominant substrate components (rubble and sand)

Hard coral vs. rubble

```
plot(Percentage[Substrate=="Hard Coral"]~Percentage[Substrate=="Rubble"], data=Benthic)
abline(lm(Percentage[Substrate=="Hard Coral"]~Percentage[Substrate=="Rubble"],
data=Benthic))
cor.test(Benthic$Percentage[Benthic$Substrate=="Hard Coral"],
Benthic$Percentage[Benthic$Substrate=="Rubble"], method="spearman")
```

```
## Warning in cor.test.default(Benthic$Percentage[Benthic$Substrate == "Hard Coral"],
## Benthic$Percentage[Benthic$Substrate == : Cannot compute exact p-value with ties
##
##  Spearman's rank correlation rho
##
## ## S = 42883.26, p-value = 1.551e-05
## alternative hypothesis: true rho is not equal to 0
## sample estimates:
##        rho
## -0.5470152
```

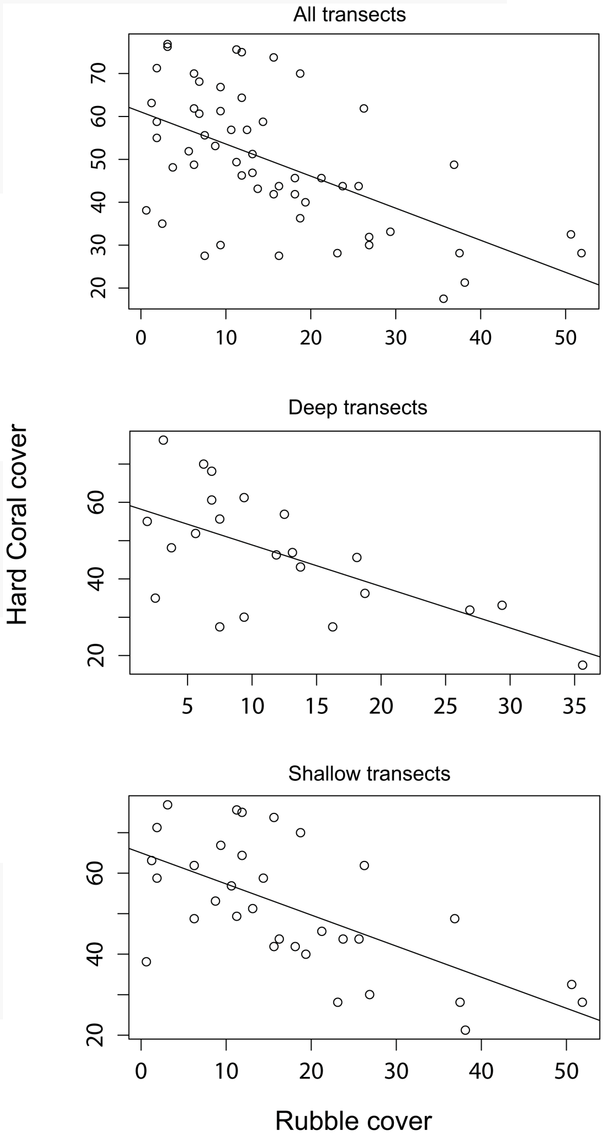

Deep transects

```
## S = 9644.2, p-value = 0.004589
## alternative hypothesis: true rho is not equal to 0
## sample estimates:
##        rho
## -0.5808367
```

Shallow transects

```
## S = 9644.2, p-value = 0.0001555
## alternative hypothesis: true rho is not equal to 0
## sample estimates:
##        rho
## -0.6116644
```

There is a statistically significant negative correlation between hard coral and rubble in the TMP (across shallow and deep transects).

Hard coral vs. sand

```
plot(Percentage[Substrate=="Hard Coral"]~Percentage[Substrate=="Sand"], data=Benthic)
abline(lm(Percentage[Substrate=="Hard Coral"]~Percentage[Substrate=="Sand"],
data=Benthic))
cor.test(Benthic$Percentage[Benthic$Substrate=="Hard Coral"],
Benthic$Percentage[Benthic$Substrate=="Sand"], method="spearman")
```

```
## Warning in cor.test.default(Benthic$Percentage[Benthic$Substrate == "Hard Coral"],
## Benthic$Percentage[Benthic$Substrate == : Cannot compute exact p-value with ties
```

```
##
##  Spearman's rank correlation rho
##
## S = 44063.26, p-value = 2.177e-06
## alternative hypothesis: true rho is not equal to 0
## sample estimates:
##        rho
## -0.5895838
```

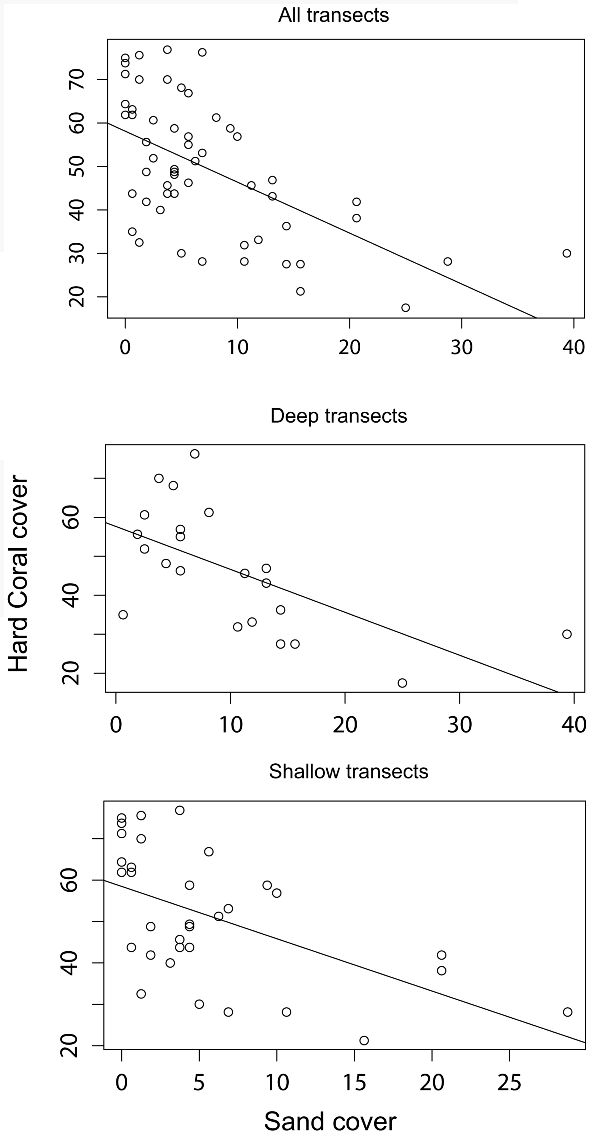

Deep transects

```
## S = 2941.145, p-value = 0.0008161
## alternative hypothesis: true rho is not equal to 0
## sample estimates:
##        rho
## -0.6607253
```

Shallow transects

```
## S = 9559.543, p-value = 0.0002411
## alternative hypothesis: true rho is not equal to 0
## sample estimates:
##        rho
## -0.5975172
```

There is a statistically significant negative correlation between hard coral and sand in the TMP (across shallow and deep transects).
