# Supplementary material for: Coral Reefs at the Northernmost Tip of Borneo: An Assessment of Scleractinian Species Richness Patterns and Benthic Reef Assemblages
Source: PLoS One. 2015 Dec 31;10(12):e0146006. doi: 10.1371/journal.pone.0146006 (PMC4697805; doi:10.1371/journal.pone.0146006)
Supplement: S7 Fig — Examining the effect of depth on the benthic communities. (PDF) [file pone.0146006.s007.pdf]

S7 Fig. Model 3. Examining the effect of depth on the benthic communities

Data was transformed using the box-cox transformation.
Substrate category silt and values of 0 percentage cover were excluded in order for the analyses to perform better.

```
boxCox(Percentage~Substrate*Depth, lambda = seq(0, 0.5, 1/10),
data=Benthic[Benthic$Percentage>0 & Benthic$Substrate!="Silt",])
```

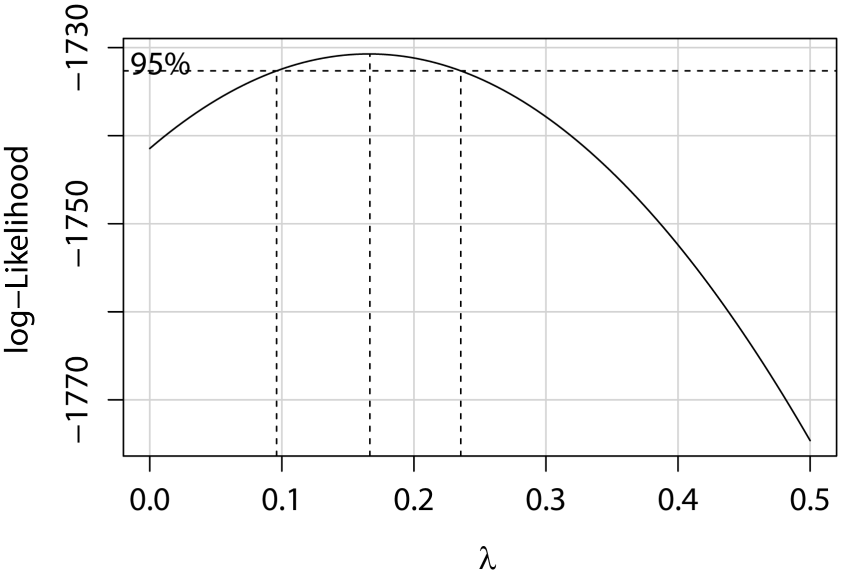

Suggested power transformation= 0.2

```
library(car)

model_depth<-lm(Percentage^(0.2)~Substrate*Depth, data=Benthic[Benthic$Percentage>0 &
Benthic$Substrate!="Silt",])


summary(model_depth)

##
## Call:
## lm(formula = Percentage^(0.2) ~ Substrate * Depth, data= Benthic[Benthic$Percentage >
##     0 & Benthic$Substrate != "Silt", ])
##
## Residuals:
##      Min       1Q   Median       3Q      Max
## -0.77277 -0.13559 -0.00835  0.12046  0.71960
##
## Coefficients:
##                                            Estimate Std. Error t value  Pr(>|t|)
## (Intercept)                                 2.13554    0.04700  45.434  < 2e-16 ***
## SubstrateNutrient Indicator Algae          -0.68141    0.06726 -10.131  < 2e-16 ***
## SubstrateOther                             -1.03725    0.08723 -11.890  < 2e-16 ***
## SubstrateRecently Killed Coral             -1.08588    0.10923  -9.942  < 2e-16 ***
## SubstrateRock                              -0.43879    0.06647  -6.601 1.43e-10 ***
## SubstrateRubble                            -0.55639    0.06647  -8.370 1.21e-15 ***
## SubstrateSand                              -0.63351    0.06647  -9.530  < 2e-16 ***
```

```
## SubstrateSoft Coral                          -0.87264    0.06811 -12.811  < 2e-16 ***
## SubstrateSponge                              -1.08084    0.07382 -14.641  < 2e-16 ***
## DepthShallow                                  0.04460    0.06068   0.735  0.46276
## SubstrateNutrient Indicator Algae:DepthShal -0.28597    0.09326  -3.066  0.00233 **
## SubstrateOther:DepthShallow                  -0.02984    0.10401  -0.287  0.77434
## SubstrateRecently Killed Coral:DepthShallow -0.03516    0.12901  -0.273  0.78534
## SubstrateRock:DepthShallow                   -0.00121    0.08582  -0.014  0.98876
## SubstrateRubble:DepthShallow                  0.05929    0.08582   0.691  0.49004
## SubstrateSand:DepthShallow                   -0.19104    0.08733  -2.187  0.02934 *
## SubstrateSoft Coral:DepthShallow             -0.13329    0.09243  -1.442  0.15011
## SubstrateSponge:DepthShallow                 -0.05344    0.09671  -0.553  0.58090
##
## ---
## Signif. codes:  0 '***' 0.001 '**' 0.01 '*' 0.05 '.' 0.1 ' ' 1
##
## Residual standard error: 0.2205 on 368 degrees of freedom
## Multiple R-squared:  0.7371, Adjusted R-squared:  0.7249
## F-statistic: 60.68 on 17 and 368 DF,  p-value: < 2.2e-16
```

There was a difference in nutrient indicator algae and sand between the shallow and deep transects. This can be seen in the boxplot of Fig. 7.

Examining the model for heteroscedacity, non-linearity and distribution of the residuals

```
qqPlot(model_depth)
residualPlots(model_depth)
## Warning in residualPlots.default(model, ...): No possible lack-of-fit tests
```

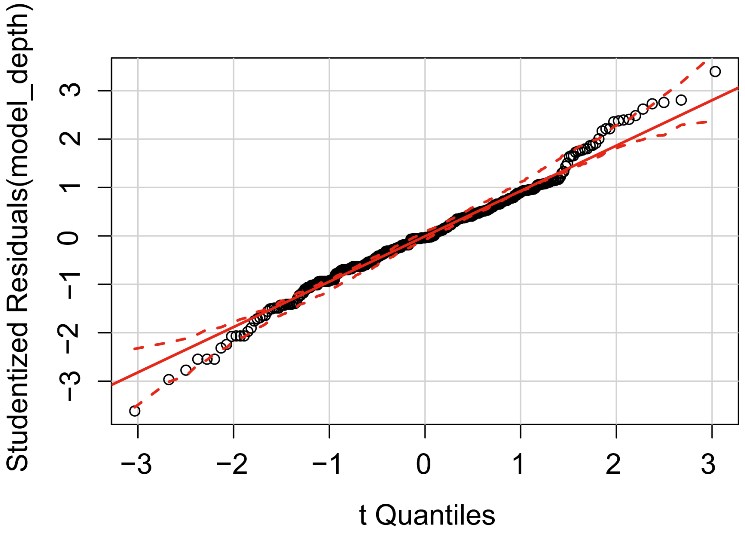

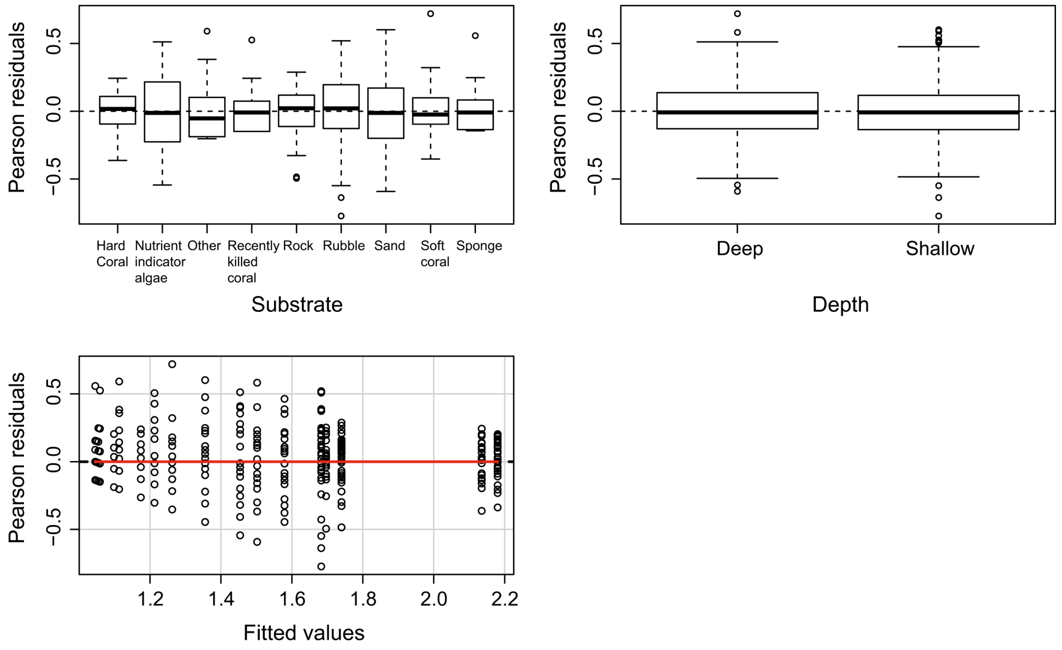


```
ncvTest(model_depth)
## Non-constant Variance Score Test
## Variance formula: ~ fitted.values
## Chisquare = 0.09966193    Df = 1     p = 0.7522357
```

```r
influenceIndexPlot(model_depth)
```

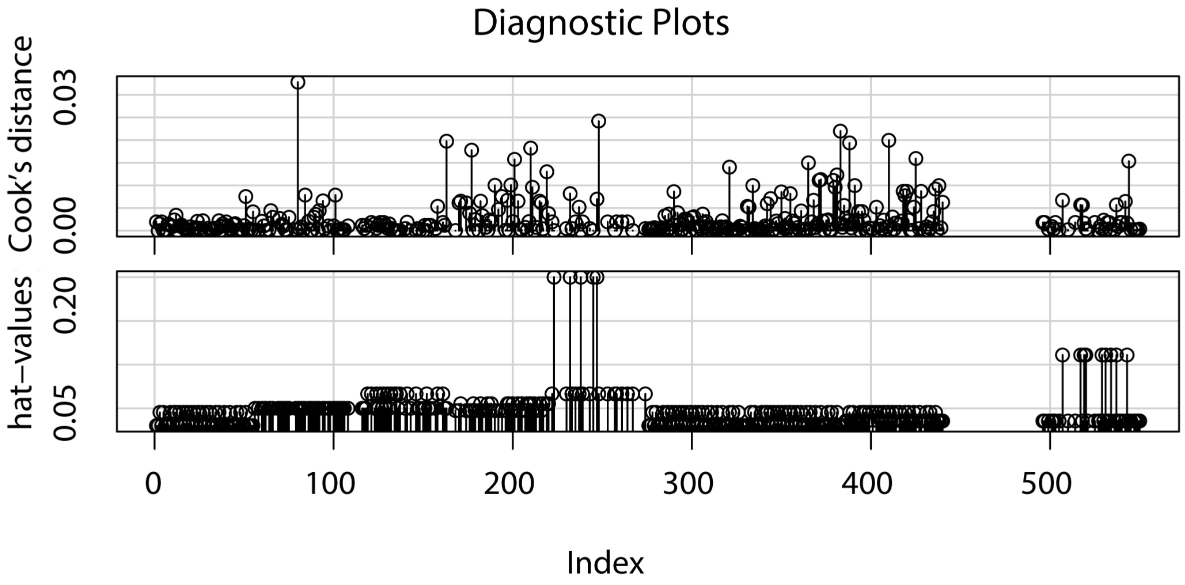


```r
outlierTest(model_depth)

## No Studentized residuals with Bonferonni p < 0.05
## Largest |rstudent|:
##      rstudent unadjusted p-value Bonferonni p
## 383 -3.617502         0.00033911       0.1309
```

The data looks fine in terms of diagnostics. The residuals are normally distributed, the variance is homogeneous and there are no outliers with Cooks distance larger than 0.1.
