# Supplementary material for: Coral Reefs at the Northernmost Tip of Borneo: An Assessment of Scleractinian Species Richness Patterns and Benthic Reef Assemblages
Source: PLoS One. 2015 Dec 31;10(12):e0146006. doi: 10.1371/journal.pone.0146006 (PMC4697805; doi:10.1371/journal.pone.0146006)

S8 Fig. Model 4. Examining the effect of distance from the mainland on the benthic communities

```
library(car)

model_dist<-lm((Percentage)^0.2~Substrate*Distance,
data=Benthic[Benthic$Substrate!="Silt" & Benthic$Percentage>0,])

summary(model_dist)

##
## Call:
## lm(formula = (Percentage)^0.2 ~ Substrate * Distance, data =
##     Benthic[Benthic$Substrate !="Silt" & Benthic$Percentage > 0, ])
##
## Residuals:
##      Min       1Q   Median       3Q      Max
## -0.88239 -0.14309  0.00333  0.12753  0.74953
##
## Coefficients:
##                                           Estimate  Std. Error t value   Pr(>|t|)
## (Intercept)                              2.0885511   0.0555334  37.609    < 2e-16 ***
## SubstrateNutrient Indicator Algae       -0.8035266   0.0854870  -9.399    < 2e-16 ***
## SubstrateOther                          -0.9614876   0.0835818 -11.504    < 2e-16 ***
## SubstrateRecently Killed Coral          -1.0350392   0.1187116  -8.719    < 2e-16 ***
## SubstrateRock                           -0.3689629   0.0785361  -4.698   3.72e-06 ***
## SubstrateRubble                         -0.2908140   0.0785361  -3.703   0.000246 ***
## SubstrateSand                           -0.5322722   0.0794600  -6.699   7.90e-11 ***
## SubstrateSoft Coral                     -0.9024140   0.894820  -10.085    < 2e-16 ***
## SubstrateSponge                         -0.9910447   0.0943909 -10.499    < 2e-16 ***
## Distance                                 0.0029894   0.0019022   1.572   0.116926
## SubstrateNutrient Indicator Algae:Dis -0.0006865   0.0028923  -0.237   0.812512
## SubstrateOther:Distance                 -0.0037063   0.0028140  -1.317   0.188628
## SubstrateRecently Killed Coral:Distan -0.0028776   0.0037251  -0.772   0.440323
## SubstrateRock:Distance                  -0.0028594   0.0026902  -1.063   0.288522
## SubstrateRubble:Distance                -0.0093221   0.0026902  -3.465   0.000592 ***
## SubstrateSand:Distance                  -0.0085808   0.0027250  -3.149   0.001772 **
## SubstrateSoft Coral:Distance            -0.0017695   0.0029784  -0.594   0.552790
## SubstrateSponge:Distance                -0.0048103   0.0031702  -1.517   0.130034
##
## ---
## Signif. codes:  0 '***' 0.001 '**' 0.01 '*' 0.05 '.' 0.1 ' ' 1
##
## Residual standard error: 0.2202 on 368 degrees of freedom
## Multiple R-squared:  0.7378, Adjusted R-squared:  0.7257
## F-statistic: 60.92 on 17 and 368 DF,  p-value: < 2.2e-16
```

Rubble and rock cover decreased with the distance from the mainland.

Examining the model for heteroscedacity, non-linearity and distribution of the residuals

```r
qqPlot(model_dist)
residualPlots(model_dist)
```

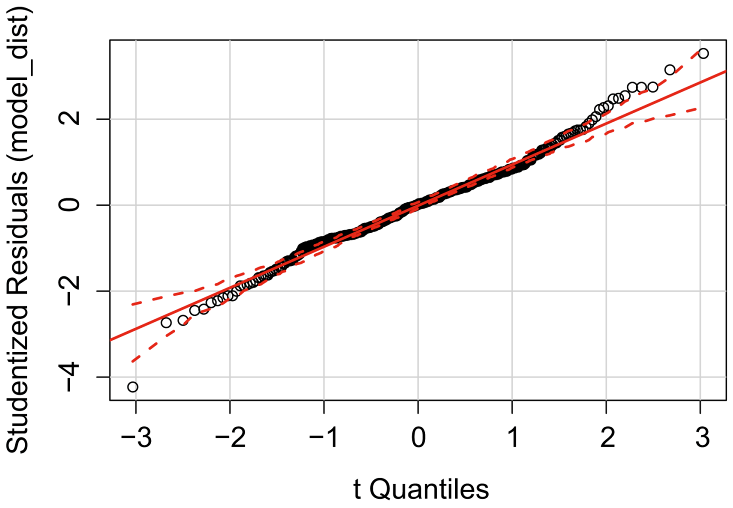

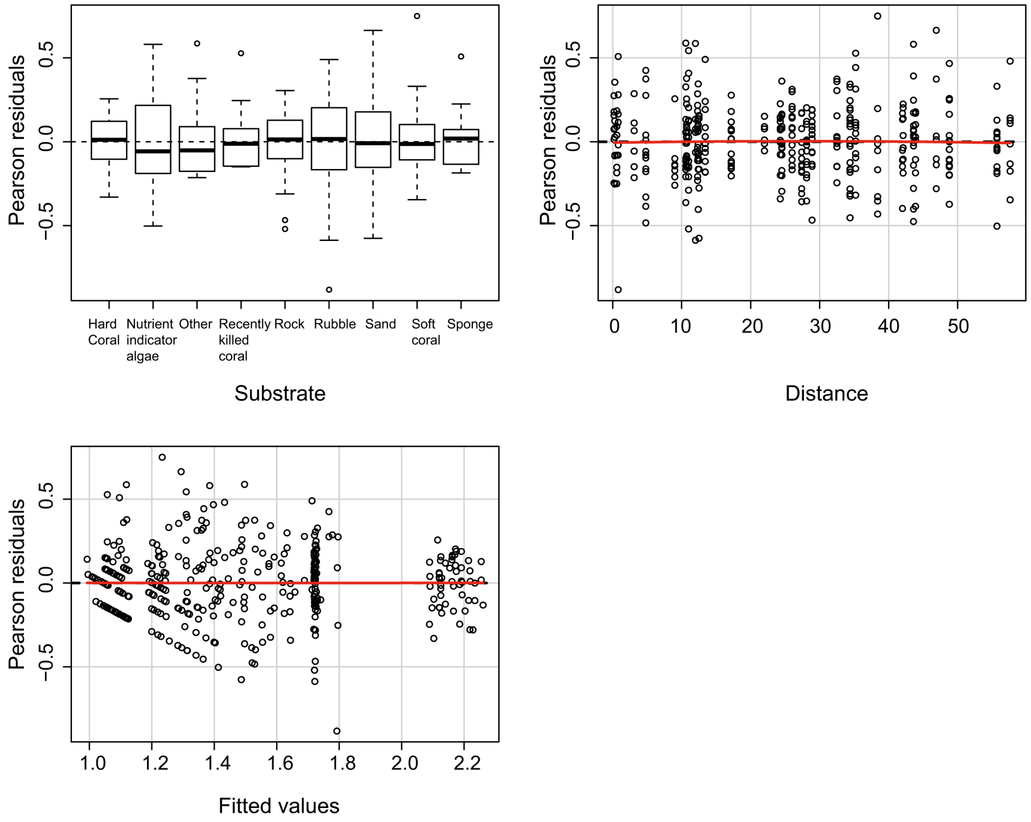

```
##              Test stat Pr(>|t|)
## Type                NA       NA
## Distance         0.266    0.790
## Tukey test       0.466    0.641

ncvTest(model_dist)
## Non-constant Variance Score Test
## Variance formula: ~ fitted.values
## Chisquare = 0.5733572    Df = 1     p = 0.4489279
```

```
influenceIndexPlot(model_dist, vars= c("Cook", "hat"))
```

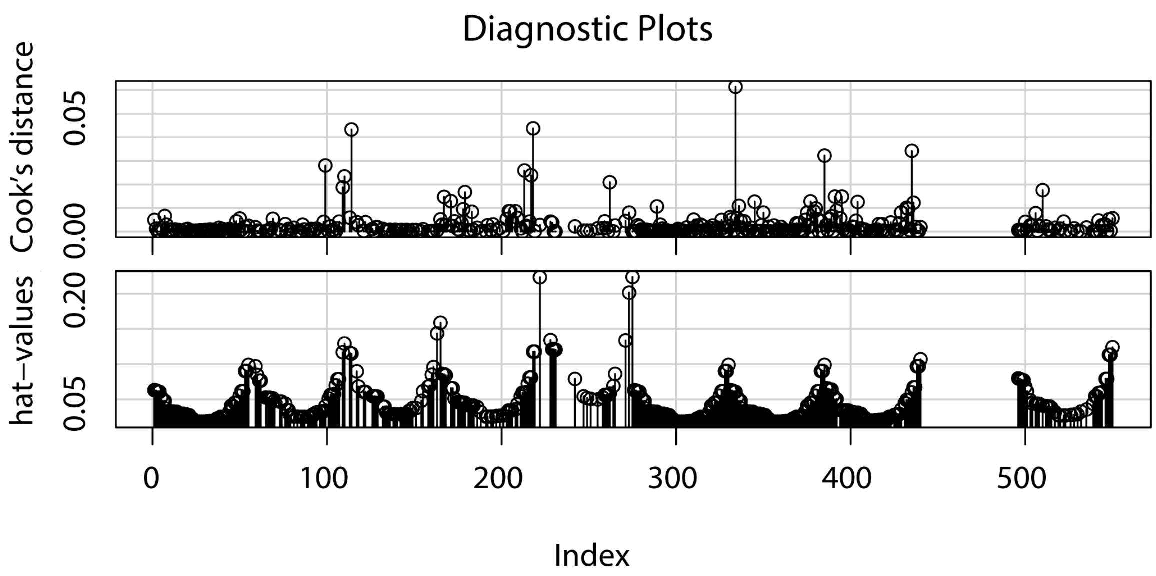


```
outlierTest(model_dist)
##      rstudent unadjusted p-value Bonferonni p
## 334 -4.229405         2.9622e-05     0.011434
```

Based on the diagnostic plots, Cook's distance are < 0.5 and most Hat-values (leverage values) are between 0.05 and 0.15. There was one outlier (observation 202).

```
Dist$Site[334]
## [1] 51S
## 55 Levels: 10D 10S 11D 11S 11S 12D 12D 13S 14D 14S 15D 16D 16S 17D 17S 18D ... 9S
```

The outlier is site 51S. Because the hat value is small, we will not exclude this site from the analysis.

The residuals are normally distributed, the variance is homogeneous and there are no outliers with Cooks distance larger than 0.1.

Similar findings were attained when the analysis was carried out separately for deep and shallow transects, that is rubble and rock cover decreased with the distance from the mainland.

Deep transects only

```
model_dist_deep<-lm((Percentage)^0.2~Substrate*Distance, data=Benthic[Benthic$Depth==
"Deep" & Benthic$Substrate!="Silt" & Benthic$Percentage>0,])

summary(model_dist_deep)

##
## Call:
## lm(formula = (Percentage)^0.2 ~ Substrate * Distance, data = Benthic[Benthic$Depth==
##     "Deep" & Benthic$Substrate !="Silt" & Benthic$Percentage > 0, ])
##
## Residuals:
##      Min       1Q   Median       3Q      Max
## -0.58016 -0.12941  0.01358  0.10226  0.67894
##
## Coefficients:
##                                           Estimate  Std. Error t value  Pr(>|t|)
## (Intercept)                               2.0197165   0.1000073  20.196   < 2e-16 ***
## SubstrateNutrient Indicator Algae        -0.7155516   0.1421270  -5.035  1.45e-06 ***
## SubstrateOther                           -0.9046430   0.2006941  -4.508  1.37e-05 ***
## SubstrateRecently Killed Coral           -0.9911070   0.3105744  -3.191  0.001749 **
## SubstrateRock                            -0.3411246   0.1414316  -2.412  0.017163 *
## SubstrateRubble                          -0.1517675   0.1414316  -1.073  0.285081
## SubstrateSand                            -0.1963324   0.1414316  -1.388  0.167288
## SubstrateSoft Coral                      -0.8658372   0.1451859  -5.964  1.91e-08 ***
## SubstrateSponge                          -0.8925802   0.1696957  -5.260  5.28e-07 ***
## Distance                                  0.0042043   0.0032612   1.289  0.199464
## SubstrateNutrient Indicator Algae:Dist 0.0012109   0.0046149   0.262  0.793412
## SubstrateOther:Distance                  -0.0046879   0.0056448  -0.830  0.407680
## SubstrateRecently Killed Coral:Distan -0.0035038   0.0098448  -0.356  0.722448
## SubstrateRock:Distance                   -0.0035449   0.0046120  -0.769  0.443418
## SubstrateRubble:Distance                 -0.0146869   0.0046120  -3.184  0.001788 **
## SubstrateSand:Distance                   -0.0158686   0.0046120  -3.441  0.000765 ***
## SubstrateSoft Coral:Distance             -0.0003062   0.0046992  -0.065  0.948139
## SubstrateSponge:Distance                 -0.0069410   0.0057817  -1.201  0.231970
##
## ---
## Signif. codes:  0 '***' 0.001 '**' 0.01 '*' 0.05 '.' 0.1 ' ' 1
##
## Residual standard error: 0.206 on 140 degrees of freedom
## Multiple R-squared:  0.7487, Adjusted R-squared:  0.7182
## F-statistic: 24.54 on 17 and 140 DF,  p-value: < 2.2e-16
```

Examining the model for heteroscedacity, non-linearity and distribution of the residuals

```
qqPlot(model_dist_deep)
residualPlots(model_dist_deep)
```

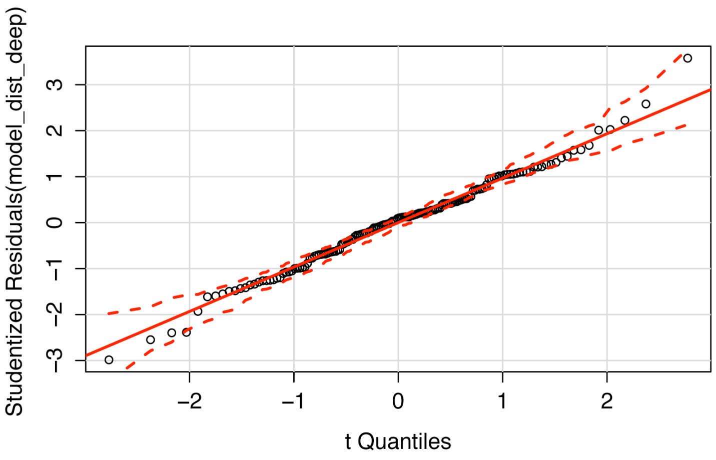

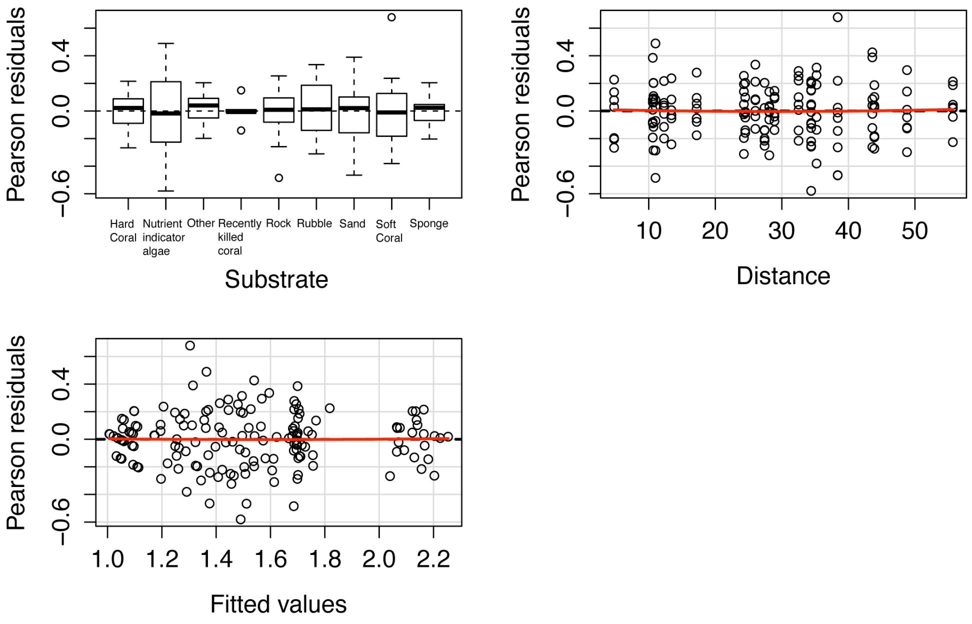


```
##              Test stat Pr(>|t|)
## Type                NA       NA
## Distance         0.252    0.801
## Tukey test       1.166    0.2414

ncvTest(model_dist_deep)
## Non-constant Variance Score Test
## Variance formula: ~ fitted.values
## Chisquare = 0.127424    Df = 1     p = 0.7211181
```

```r
influenceIndexPlot(model_dist_deep, vars= c("Cook", "hat"))
```

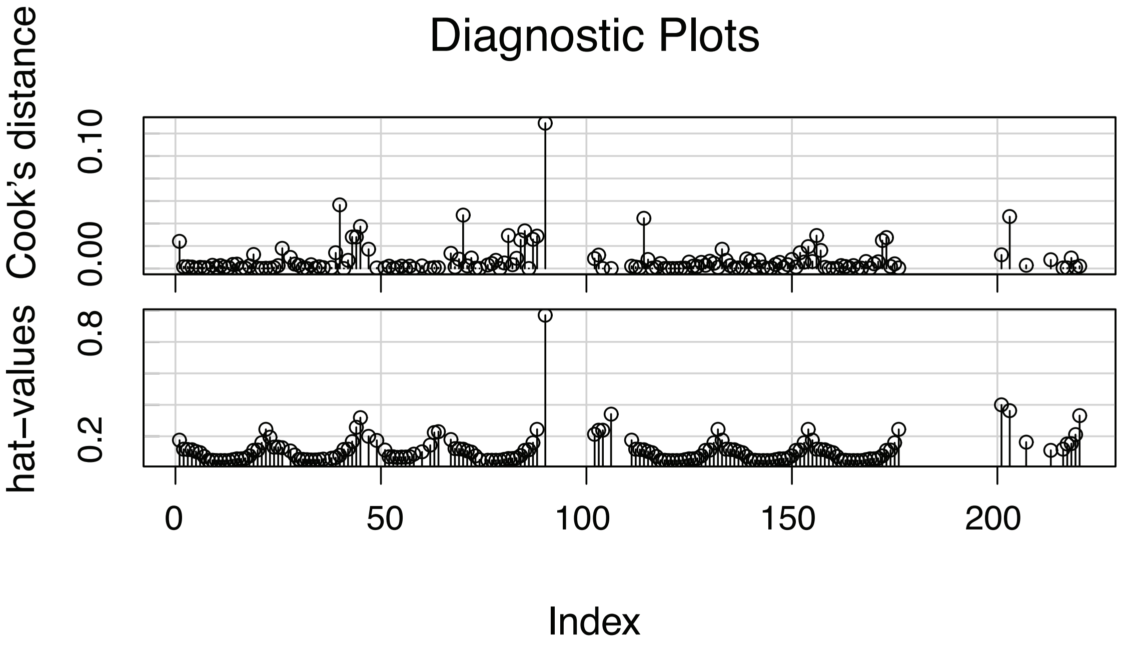

Shallow transects only

```r
model_dist_shallow<-lm((Percentage)^0.2~Substrate*Distance, data=Benthic[Benthic$Depth==
"Shallow" & Benthic$Substrate!="Silt" & Benthic$Percentage>0,])

summary(model_dist_shallow)

##
## Call:
## lm(formula = (Percentage)^0.2 ~ Substrate * Distance, data = Benthic[Benthic$Depth==
##     "Shallow" & Benthic$Substrate !="Silt" & Benthic$Percentage > 0, ])
##
## Residuals:
##      Min       1Q   Median       3Q      Max
##  -0.8606  -0.1402   0.0042   0.1174   0.7067
##
## Coefficients:
##                                         Estimate   Std. Error t value  Pr(>|t|)
## (Intercept)                             2.114920     0.065370  32.353   < 2e-16 ***
## SubstrateNutrient Indicator Algae      -0.859800     0.104891  -8.197  2.41e-14 ***
## SubstrateOther                         -0.986615     0.093721 -10.527   < 2e-16 ***
## SubstrateRecently Killed Coral         -1.057416     0.130376  -8.111  4.16e-14 ***
## SubstrateRock                          -0.379409     0.092447  -4.104  5.81e-05 ***
## SubstrateRubble                        -0.340815     0.092447  -3.687  0.000289 ***
## SubstrateSand                          -0.667873     0.093981  -7.106  1.83e-11 ***
## SubstrateSoft Coral                    -0.914523     0.111527  -8.200  2.37e-14 ***
## SubstrateSponge                        -1.029190     0.112357  -9.160   < 2e-16 ***
```

```
## Distance                                  0.002867     0.002321   1.235  0.218098
## SubstrateNutrient Indicator Algae:Dis -0.004793     0.003697  -1.296  0.196331
## SubstrateOther:Distance               -0.003583     0.003387  -1.058  0.291389
## SubstrateRecently Killed Coral:Distan -0.002810     0.004121  -0.682  0.496027
## SubstrateRock:Distance                -0.002663     0.003282  -0.811  0.418096
## SubstrateRubble:Distance              -0.006868     0.003282  -2.093  0.037565 *
## SubstrateSand:Distance                -0.007049     0.003355  -2.101  0.036862 *
## SubstrateSoft Coral:Distance          -0.003906     0.003793  -1.030  0.304343
## SubstrateSponge:Distance              -0.004392     0.003745  -1.173  0.242228
##
## ---
## Signif. codes:  0 '***' 0.001 '**' 0.01 '*' 0.05 '.' 0.1 ' ' 1
##
## Residual standard error: 0.2214 on 210 degrees of freedom
## Multiple R-squared:  0.7674, Adjusted R-squared:  0.7486
## F-statistic: 40.76 on 17 and 210 DF,  p-value: < 2.2e-16
```

Examining the model for heteroscedacity, non-linearity and distribution of the residuals

```r
qqPlot(model_dist_shallow)
residualPlots(model_dist_shallow)
```

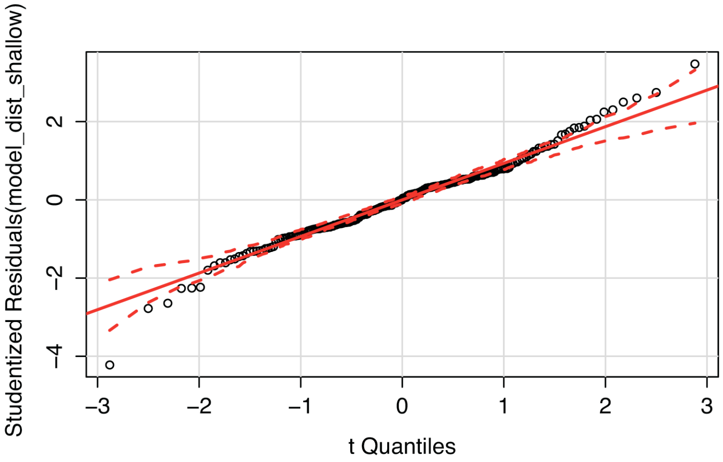

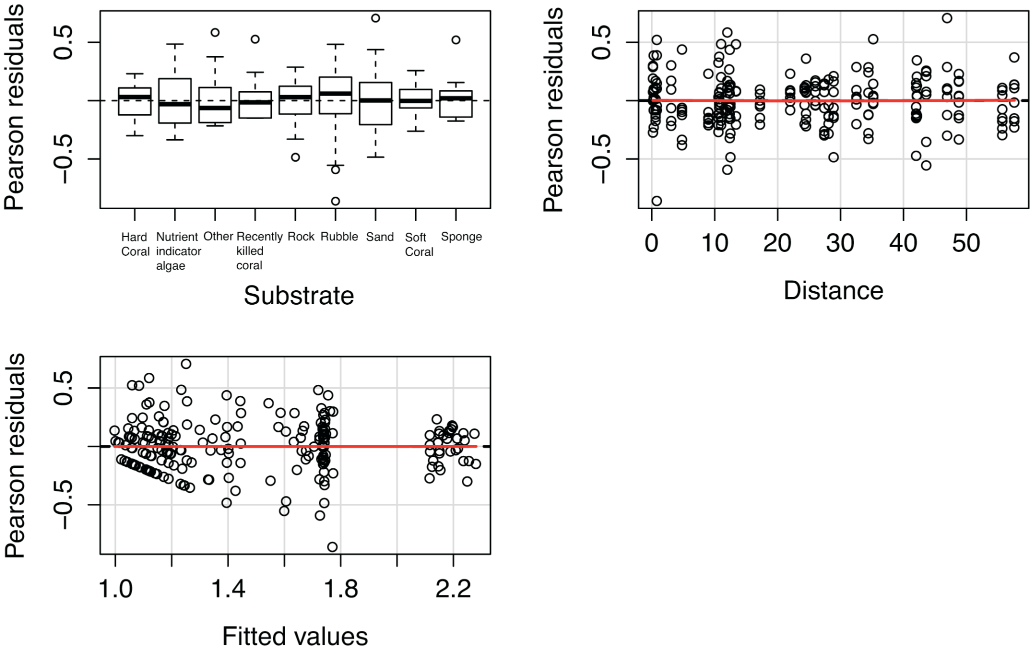


```
##            Test stat Pr(>|t|)
## Type              NA       NA
## Distance       0.113    0.910
## Tukey test     0.548    0.584

ncvTest(model_dist_shallow)
## Non-constant Variance Score Test
## Variance formula: ~ fitted.values
## Chisquare = 0.06799472    Df = 1     p = 0.7942792
```

```r
influenceIndexPlot(model_dist_shallow, vars= c("Cook", "hat"))
```

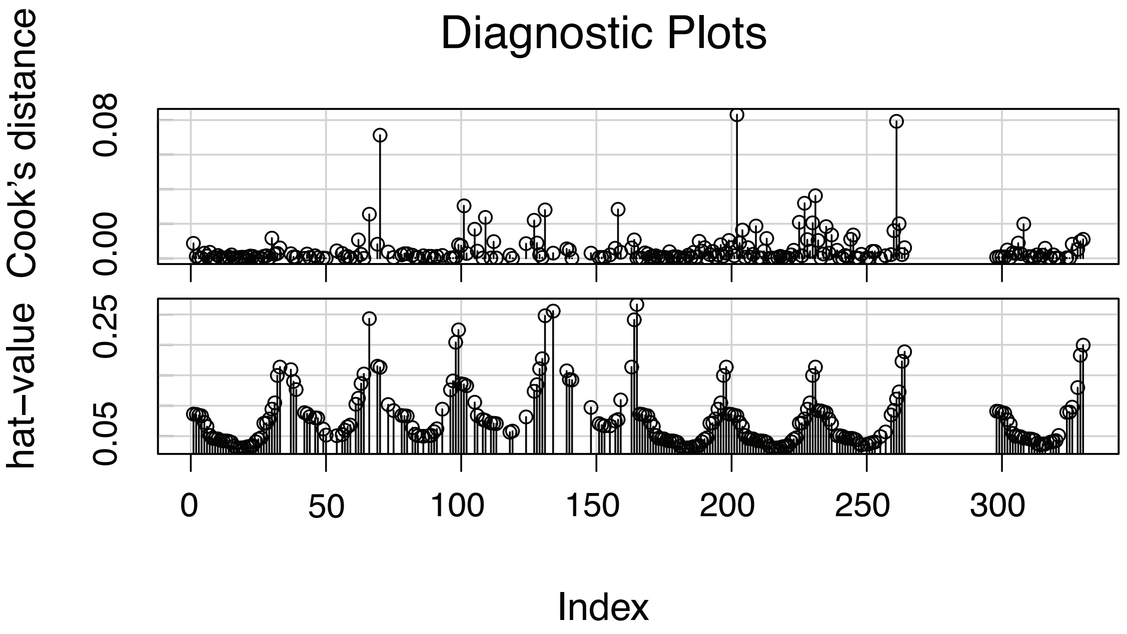

Supplement: S8 Fig — Examining the effect of distance from the mainland on the benthic communities. (PDF) [file pone.0146006.s008.pdf]
