# Supplementary figures and images for: Coral Reefs at the Northernmost Tip of Borneo: An Assessment of Scleractinian Species Richness Patterns and Benthic Reef Assemblages
Source: PLoS One. 2015 Dec 31;10(12):e0146006. doi: 10.1371/journal.pone.0146006 (PMC4697805; doi:10.1371/journal.pone.0146006)

**S9 Fig. *Pavona* spp.** (A) *Pavona* sp. 1 from site 53, (B) *Pavona* sp. 2 from site 3.

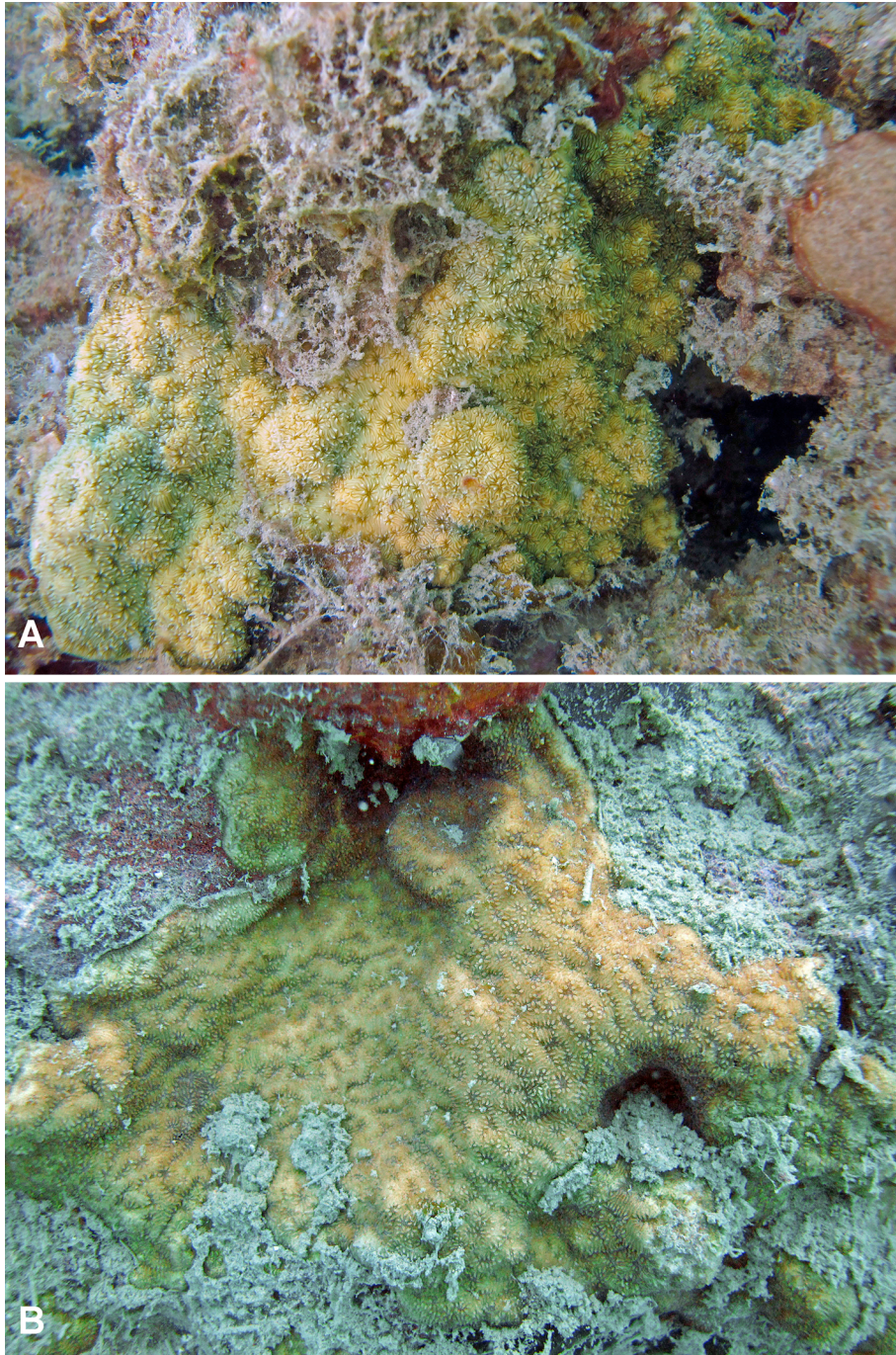

Supplement: S9 Fig — (PDF) [file pone.0146006.s009.pdf]
